# Genome-Wide Characterization of PX Domain-Containing Proteins Involved in Membrane Trafficking-Dependent Growth and Pathogenicity of Fusarium graminearum

**DOI:** 10.1128/mBio.02324-21

**Published:** 2021-12-21

**Authors:** Yi Lou, Jing Zhang, Guanghui Wang, Wenqin Fang, Shumin Wang, Yakubu Saddeeq Abubakar, Jie Zhou, Zonghua Wang, Wenhui Zheng

**Affiliations:** a State Key Laboratory of Ecological Pest Control for Fujian and Taiwan Crops, College of Life Sciences, Fujian Agriculture and Forestry University, Fuzhou, China; b Key Laboratory of Bio-pesticide and Chemistry Biology, Ministry of Education, College of Plant Protection, Fujian Agriculture and Forestry University, Fuzhou, Fujian, China; c College of Plant Protection, Northwest A&F University, Yangling, China; d Department of Biochemistry, Faculty of Life Sciences, Ahmadu Bello University, Zaria, Nigeria; e Institute of Oceanography, Minjiang University, Fuzhou, China; Yonsei University

**Keywords:** PX domain, FgBem1, septum development, pathogenicity, *Fusarium graminearum*, PX domain

## Abstract

The Phox homology (PX) domain is a membrane recruitment module that binds to phosphoinositides (PI) mediating the selective sorting and transport of transmembrane proteins, lipids, and other critical cargo molecules via membrane trafficking processes. However, the mechanism of vesicular trafficking mediated by PX domain-containing proteins in phytopathogenic fungi and how this relates to the fungal development and pathogenicity remain unclear. Here, we systematically identified and characterized the functions of PX domain-containing proteins in the plant fungal pathogen Fusarium graminearum. Our data identified 14 PX domain-containing proteins in F. graminearum, all of which were required for plant infection and deoxynivalenol (DON) production, with the exception of FgMvp1 and FgYkr078. Furthermore, all the PX domain-containing proteins showed distinct localization patterns that were limited to the endosomes, vacuolar membrane, endoplasmic reticulum, cytoplasm, and hyphal septa/tips. Remarkably, among these proteins, FgBem1 targeted to surface crescent and septal pores and was retained at the septum pores even after actin constriction during septum development. Further analyses demonstrated that the surface crescent targeting of FgBem1 solely depended on its SH3 domains, while its septum and apex anchoring localization relied on its PX domain, which was also indispensable for reactive oxygen species (ROS) production, sexual development, and pathogenicity in F. graminearum. In summary, our study is the first detailed and comprehensive functional analysis of PX domain-containing proteins in filamentous fungi, and it provides new insight into the mechanism of FgBem1 involved in septum and apex anchorage mediated by its PX domain, which is necessary for sexual development and pathogenicity of F. graminearum.

## INTRODUCTION

Fusarium graminearum is a filamentous ascomycete that causes a devastating disease of cereal crops and is the predominant causal agent of most severe wheat diseases worldwide (1, 2). Practically, the epidemic of Fusarium head blight (FHB) gives rise to severe wheat yield losses in China (3, 4). Besides the yield loss, F. graminearum produces harmful mycotoxins, such as zearalenone (ZEA) and deoxynivalenol (DON) in infected grains, which are detrimental to the health of human and livestock (5, 6). DON, as an inhibitor of protein synthesis in eukaryotic organisms, is an important virulence factor during plant infection. To date, managing FHB has been difficult because of the lack of resistant cultivars and effective fungicides. Therefore, improving our understanding of the genetic mechanism of F. graminearum pathogenesis will facilitate the development of efficient management strategies against this devastating pathogen.

The Phox homology (PX) domain is a membrane recruitment module that binds to phosphoinositides (PI) on the cytoplasmic leaflets of different organelles. It is primarily involved in the selective sorting and transport of transmembrane proteins, lipids, and other critical cargo molecules via membrane trafficking processes (7). Specially, the PX domain has been reported to bind phosphatidic acid, phosphatidylinositol-3-bisphosphate [PI(3)P], phosphatidylinositol-3,4-bisphosphate [PI(3,4)P2], phosphatidylinositol-3,5-bisphosphate [PI(3,5)P2], phosphatidylinositol-4,5-bisphosphate [PI(4,5)P2], and phosphatidylinositol-3,4,5-triphosphate [PI(3,4,5)P3] (7, 8). So far, more than 100 PX domain-containing proteins have been identified in eukaryotes from yeast to human. There are 15 PX domain-containing proteins in yeast, whereas mammals have as much as 49 (9, 10). Based on their conserved domains and functions, most PX domain-containing proteins are classified as sorting nexins (SNXs) subfamily, which mediates the retrieval of membrane proteins within the endocytic and secretory pathways (9).

In mammalian cells, two distinct PX domain-containing proteins, Snx17 and Snx27, have been identified as critical regulators of recycling from endosomes to the cell surface via the interaction with cargo receptors and Ras GTPases (11). Deletion of *SNX8* significantly increased the bacterial Shiga toxin (Stx) transport to the *trans*-Golgi network (TGN), whereas the transport of the plant toxin ricin was slightly inhibited, indicating a critical role of Snx8 in the retrograde transport of cargo molecules (12). Also, Snx13, a member of the sorting nexin and a regulator of G protein signaling (RGS), mediates heart failure through degradative sorting of the apoptosis repressor (13). In Saccharomyces cerevisiae, Mvp1 (a ortholog of Snx8) has been suggested to function in anterograde protein transport from the Golgi to the endosome (14), while Vps5 (Snx1 ortholog), Vps17 (Snx2 ortholog), and Grd19 (Snx3 ortholog) are required for the retrieval of proteins such as Kex2 and Vps10 from endosomal compartments (15–18). Further study demonstrated that Mdm1 (a homolog of Snx13 in yeast), as a novel interorganelle-tethering protein, may play previously unappreciated roles in interorganelle communication and lipid metabolism (19). In addition, Snx4 and Snx42 (also known as ATG20) are involved in the endosomal sorting of the protein Ape1 (a cytosolic hydrolase) through the cytoplasm-to-vacuole targeting pathway (20).

Compared to the yeasts and mammalians, the functional studies of SNXs subfamily in filamentous fungi are relatively few. In F. graminearum, FgRgsC (Snx13 ortholog) was identified as an RGS protein and required for the discharge of ascospores from perithecia (21). We previously revealed that the two subunits of the retromer complex Vps5 and Vps17 (Snx1 and Snx2 orthologs, respectively) are required for vesicle trafficking-associated growth, sexual development, and pathogenicity of F. graminearum (22). Subsequently, we further found that the sorting nexins, FgSnx41 and FgSnx4, assemble into a functionally interdependent heterodimer and mediate FgSnc1 recycling, which facilitates fungal polarized growth and pathogenicity in F. graminearum (23). These studies indicate that PX domain-containing proteins are important for fungal development and pathogenicity through the membrane trafficking pathway. However, systemic functional characterizations of the PX domain-containing proteins in filamentous fungi are still lacking.

In addition to the SNX subfamily, PX domain is also present in other proteins such as Vam7 and Bem1 (24). In phytopathogens, Vam7 functions have been widely documented. In the corn smut fungus Ustilago maydis, UmYup1 (a Vam7-like protein) is known to mediate endocytic recycling through early endosomes and is required for hyphal morphogenesis and pathogenesis (25, 26). In Magnaporthe oryzae, MoVam7, as a soluble *N*-ethylmaleimide-sensitive factor attachment protein receptor (SNARE) protein homolog, is required for vacuolar morphology and endocytosis. Δ*Movam7* showed significant defects in hyphal growth, conidial formation, and virulence of M. oryzae (27). In F. graminearum, FgVam7 is a vacuolar membrane-localized protein and plays a crucial role in the development and virulence of the fungus through mediation of membrane fusion and vesicle trafficking (28–31). As a scaffold protein, Bem1 plays an important role in the Cdc42-dependent establishment of polarized growth during the cell cycle. Recruitment of Fus3 (a MAP kinase) to the shmoo (polar-growing mating projections) tip, where it activates the formin Bni1, is promoted by the scaffold protein Bem1 (32–34). Further study demonstrated that Bem1 contributes to the secretory pathway polarization through a direct interaction with Exo70p, suggesting that the spatial coupling of apical exocytosis and the endocytic recycling of polarized materials at the hyphal tip may be mediated by Bem1 (35). In Epichloë festucae, Bem1 localizes at the hyphal tips and physically interacts with Cdc24, which is involved in localizing NoxR protein to the sites of the fungal hyphal morphogenesis and growth, indicating that the protein machinery was responsible for the fungal polarity establishment and Nox complex controlling cellular differentiation (36). In Botrytis cinerea, BcBem1 is also localized in the cytoplasm and hyphal tips. The establishment and maintenance of polarity were mediated by the interaction between Bem1, Cdc24, and the formin Bni1 (37). In addition, increasing evidence supports that Bem1 protein appears as a cortical ring around the septal pore after septation in Neurospora crassa (38). However, the functions of Bem1 are yet to be established in F. graminearum.

Although PX domain-containing proteins widely exist in the eukaryotes, system-wide characterization of PX domain family proteins, particularly in subcellular localization and biological function, are poorly reported. Herein, we identified 14 PX domain-containing proteins in F. graminearum through a genome-wide *in silico* analysis. Subcellular localizations analysis revealed that they show distinct localization patterns, including endosomes, vacuolar membrane, endoplasmic reticulum, cytoplasm, and septal hyphae-specific compartments (e.g., septal pore and surface crescent of the growing hyphal tips). Characterization using gene deletion mutants revealed that all the PX domain-containing proteins except FgMvp1 and FgYKR078 are important for virulence and DON production in F. graminearum. Interestingly, we found that the PX domain of FgBem1 is important for septum and apex anchoring in growing hyphae, which is involved in septation-associated nuclear division and reactive oxygen species (ROS) generation during hyphal apical growth in F. graminearum.

## RESULTS

### Phylogenetic analysis of homologous PX domain-containing proteins in fungi.

Members of the PX domain-containing family possess a conserved PX-domain acting as a phosphoinositide-binding motif. Using InterPro terms, 52 PX domain-containing proteins were retrieved from four model filamentous fungi (Aspergillus nidulans, F. graminearum, M. oryzae, and N. crassa) and S. cerevisiae (an acknowledged model organism) genomes (https://fungidb.org/fungidb/) via BLASTP analysis. Among them, 14 annotated genes (FGSG_05261, FGSG_10187, FGSG_13543, FGSG_09917, FGSG_13137, FGSG_02586, FGSG_08932, FGSG_12183, FGSG_17200, FGSG_02011, FGSG_09600, FGSG_09157, FGSG_06950, and FGSG_16691) were found in F. graminearum (Table 1). These 14 genes were further identified as *FgBEM1*, *FgSNX19*, *FgMDM1*, *FgSPO14*, *FgYPR097*, *FgYKR078*, *FgSNX3*, *FgMVP1*, *FgYPT35*, *FgVPS5*, *FgVPS17*, *FgSNX4*, *FgSNX41*, and *FgVAM7*, respectively, based on their homology with yeast (Table 1). Next, we classified these PX domain-containing proteins into 14 categories (including groups 1 to 14) based on their possessions of different functional domains (Fig. S1 in the supplemental material). This suggests that expansions of the PX domain-containing proteins are functionally diverse. Members of group 1 are homologs of the phopholipase D family, which contained PX, pleckstrin homology (PH), and phospholipase D (PLD) domains. Members of group 2 are homologs of the SNARE protein Vamp7, which contained PX and soluble *N*-ethylmaleimide-sensitive factor attachment protein receptor domain (SNARE) domains. Members of groups 3, 6, 11, 12, and 14 contain a membrane-curvature-sensing Bin/amphiphysin/Rvs (BAR) domain and therefore are considered SNX-BAR subfamily. Each member of groups 4 and 10 contains a PX-associated domain termed PXA in addition to the PX domain. Group 5 members were homologs of Bem1 protein,s which contain four conserved domains, including the PX domain, two Src homology 3 (SH3) domains, and PB1 (a proline-rich domain mediating specific protein-protein interactions) domain. These results reveal that the PX domain-containing proteins are well conserved in filamentous fungi and have distinct roles/localizations due to domain expansion throughout evolution.

**TABLE 1:** Localizations of various PX domain-containing proteins in F. graminearum

Gene ID	Gene name	Localization	Source or reference
FGSG_05261	*FgBEM1*	Surface crescent, septal pore	This study
FGSG_10187	*FgSNX19*	Endoplasmic reticulum	This study
FGSG_13543	*FgMDM1 (FgRGSC)*	Endoplasmic reticulum	21
FGSG_09917	*FgSPO14(FgPLD1)*	Cytoplasm	62
FGSG_13137	*FgYPR097*	Cytoplasm	This study
FGSG_02586	*FgYKR078*	Cytoplasm	This study
FGSG_08932	*FgSNX3*	Early endosomes	This study
FGSG_12183	*FgMVP1*	Early and late endosomes	This study
FGSG_17200	*FgYPT35*	Vacuolar membrane	This study
FGSG_02011	*FgVPS5*	Early and late endosomes	22
FGSG_09600	*FgVPS17*	Early and late endosomes	22
FGSG_09157	*FgSNX4*	Early endosomes	23
FGSG_06950	*FgSNX41*	Early endosomes	23
FGSG_16691	*FgVAM7*	Vacuolar membrane	28 – 30

10.1128/mBio.02324-21.1 FIG S1 Phylogenetic analysis of putative PX domain-containing proteins in fungi. A neighbor-joining tree was constructed based on the amino acid sequences of representative fungal PX domain-containing proteins. Numbers at nodes represent the percentage of their occurrence in 10,000 bootstrap replicates; only nodes supported by 50 bootstraps or more are shown. The PX domain-containing proteins are separated into 14 different clades (designated groups 1 to 14). The colored boxes in the protein domain architecture represent the homeodomains. PX, Phox homology domain; PXA, PX-associated domains; RGS, regulator of G protein signaling domain; BAR, Bin/amphiphysin/Rvs (BAR) domain; PB1, proline-rich domain mediating specific protein-protein interactions; SH3, Src homology 3 domain; PLD, phospholipase D domain; PH, pleckstrin homology domain; SNARE, soluble *N*-ethylmaleimide-sensitive factor attachment protein receptor domain; RhoGAP, GTPase-activator protein (GAP) domain (Rho-like GTPases). Download FIG S1, TIF file, 0.7 MB. Copyright © 2021 Lou et al. 2021 Lou et al. https://creativecommons.org/licenses/by/4.0/ This content is distributed under the terms of the Creative Commons Attribution 4.0 International license.

### PX domain-containing proteins have distinct localization patterns in F. graminearum.

To determine the subcellular localizations of the PX domain-containing proteins in F. graminearum, we tagged each of the proteins with green fluorescent protein (GFP), respectively, under the control of their respective native promoters. The resulting transformants were confirmed by PCR using the primers indicated in Table S1C. We then observed their subcellular localizations by laser-scanning confocal microscopy. The phenotypes of all of the resulting transformants were similar to the wild-type PH-1, suggesting that GFP tagging does not affect the functions of the proteins. The subcellular localizations of these GFP-tagged proteins were observed in the fungal conidia and mycelia grown in complete medium (CM) or trichothecene biosynthesis induction (TBI) medium (Fig. S2). The results showed that the subcellular localizations of these PX domain-containing proteins were different and can be grouped into five different subcellular regions. Among them, the FgSnx4-GFP, FgSnx41-GFP, FgVps5-GFP, FgVps17-GFP, FgSnx3-GFP, and FgMvp1-GFP were found to localize to some punctate structures in the conidia and mycelia. FgYpt35-GFP was found to localize to some ring-shaped structures in both conidia and mycelia. FgYpr097-GFP and FgSpo14-GFP dispersed throughout the cell, suggesting that these proteins are localized to the cytoplasm. FgSnx19-GFP and FgMdm1-GFP were observed as some reticular structures in cells of conidia and mycelia. Interestingly, FgBem1-GFP localized to septa and conidial tips (Fig. S2; Table 2). These results indicate that PX domain-containing proteins exhibit distinct subcellular localization patterns in F. graminearum.

**TABLE 2:** Characterization of growth, conidiation, virulence, and deoxynivalenol production in F. graminearum strains

Strain	Growth^*a*^ (cm)	Conidiation^*b*^ (×10^4^ spores/ml)	Disease index^*c*^	DON^*d*^ (μg/mg)
PH-1	6.87 ± 0.18 a^*e*^	120.11 ± 7.82 a	13.2 ± 0.84 a	17.76 ± 1.85 a
Δ*Fgbem1*	5.75 ± 0.07 b	23.58 ± 7.21 d	1.8 ± 0.84 cd	2.99 ± 0.66 c
Δ*Fgbem1-C*	6.37 ± 0.25 a	105.21 ± 9.33 a	11.3 ± 1.20 a	15.64 ± 0.83 a
*FgBem1* ^ΔSH3-1^	5.43 ± 0.31 b	55.78 ± 11.31 bc	2.8 ± 1.04 cd	NA^*f*^
*FgBem1* ^ΔSH3-2^	5.56 ± 0.15 b	65.78 ± 15.32bc	1.9 ± 1.34 cd	NA
*FgBem1* ^ΔPX^	5.48 ± 0.21 b	58.78 ± 13.31 bc	2.2 ± 1.05 cd	NA
*FgBem1* ^ΔPB1^	5.51 ± 0.17 b	71.78 ± 8.31 bc	3.5 ± 1.54 cd	NA
Δ*Fgsnx3*	6.69 ± 0.14 a	88.67 ± 7.05 b	7.1 ± 1.58 b	5.16 ± 0.41 b
Δ*Fgsnx3-C*	6.51 ± 0.41 a	106.17 ± 8.34 a	12.1 ± 0.73 a	14.56 ± 1.35 ab
Δ*Fgypt35*	7.19 ± 0.51 a	94.67 ± 8.94 ab	6.2 ± 1.30 b	4.54 ± 0.93 b
Δ*Fgypt35-C*	6.89 ± 0.41 a	102.83 ± 7.98 a	11.8 ± 1.02 a	12.56 ± 0.88 ab
Δ*Fgspo14*	1.09 ± 0.08 c	17.22 ± 5.63 d	1.2 ± 0.45 d	0.57 ± 0.06 d
Δ*Fgsnx19*	6.80 ± 0.15 a	44.4 ± 13.18 c	3.6 ± 0.89 c	3.21 ± 0.79 bc
Δ*Fgsnx19-C*	6.77 ± 0.24 a	104.11 ± 8.08 a	10.86 ± 2.21 a	12.45 ± 1.51 ab
Δ*Fgypr097*	6.49 ± 0.53 a	57.67 ± 15.16 c	3.6 ± 1.52 c	2.03 ± 0.54 c
Δ*Fgypr097-C*	6.94 ± 0.41 a	108.78 ± 11.21 a	12.1 ± 1.88 a	14.88 ± 1.22 a
Δ*Fgmdm1*	7.04 ± 0.37 a	48.5 ± 16.57 c	3.2 ± 1.16 c	3.86 ± 0.77 b
Δ*Fgmdm1-C*	6.84 ± 0.56 a	104.67 ± 8.24 a	11.2 ± 1.65 a	11.45 ± 2.21 ab
Δ*Fgykr078*	6.98 ± 0.26 a	102.22 ± 6.04 a	14.4 ± 2.30 a	15.31 ± 0.99 a
Δ*Fgmvp1*	7.19 ± 0.60 a	109.67 ± 10.14 a	12.2 ± 1.30 a	15.98 ± 0.68 a

a Vegetative hyphal growth rate was measured after incubation on CM media for 3 days. Data were presented as mean ± SD from three independent experiments.

b Conidia production of each strain was measure after incubating for 4 days in carboxymethylcellulose (CMC) culture. Data are presented as mean ± SD from four independent experiments.

c Diseased spikelets per wheat head were measured at 14 days postinoculation (dpi). At least five wheat heads were examined in each repeat.

d DON production was measured in liquid trichothecene biosynthesis (LTB) cultures after incubation for 7 days at 25°C. Data are presented as mean ± SD from three independent experiments.

e Means ± SE were calculated from three independent experiments. Data were analyzed with SPSS using one-way analysis of variance (ANOVA). The same letter indicates that there was no significant difference, and different letters indicate statistically significant difference (*P* < 0.01).

f NA, not assayed.

10.1128/mBio.02324-21.2 FIG S2 Localization patterns of the PX domain-containing proteins in F. graminearum. The localizations of FgSnx4-GFP, FgSnx41-GFP, FgVps5-GFP, FgVps17-GFP, FgSnx3-GFP, FgMvp1-GFP, FgYpt35-GFP, FgYpr097-GFP, FgSpo14-GFP, FgSnx19-GFP, FgMdm1-GFP, FgYkr078-GFP, and FgBem1-GFP in the conidia or mycelia grown on CM or TBI (DON-inducing) medium were observed by fluorescence microscopy. Download FIG S2, TIF file, 2.9 MB. Copyright © 2021 Lou et al. 2021 Lou et al. https://creativecommons.org/licenses/by/4.0/ This content is distributed under the terms of the Creative Commons Attribution 4.0 International license.

10.1128/mBio.02324-21.7 TABLE S1 Strains, plasmids, and PCR primers used in this study. Download Table S1, DOCX file, 0.03 MB. Copyright © 2021 Lou et al. 2021 Lou et al. https://creativecommons.org/licenses/by/4.0/ This content is distributed under the terms of the Creative Commons Attribution 4.0 International license.

### Most PX domain-containing proteins associate with endosomes and/or the ER in F. graminearum.

**FIG 1:**
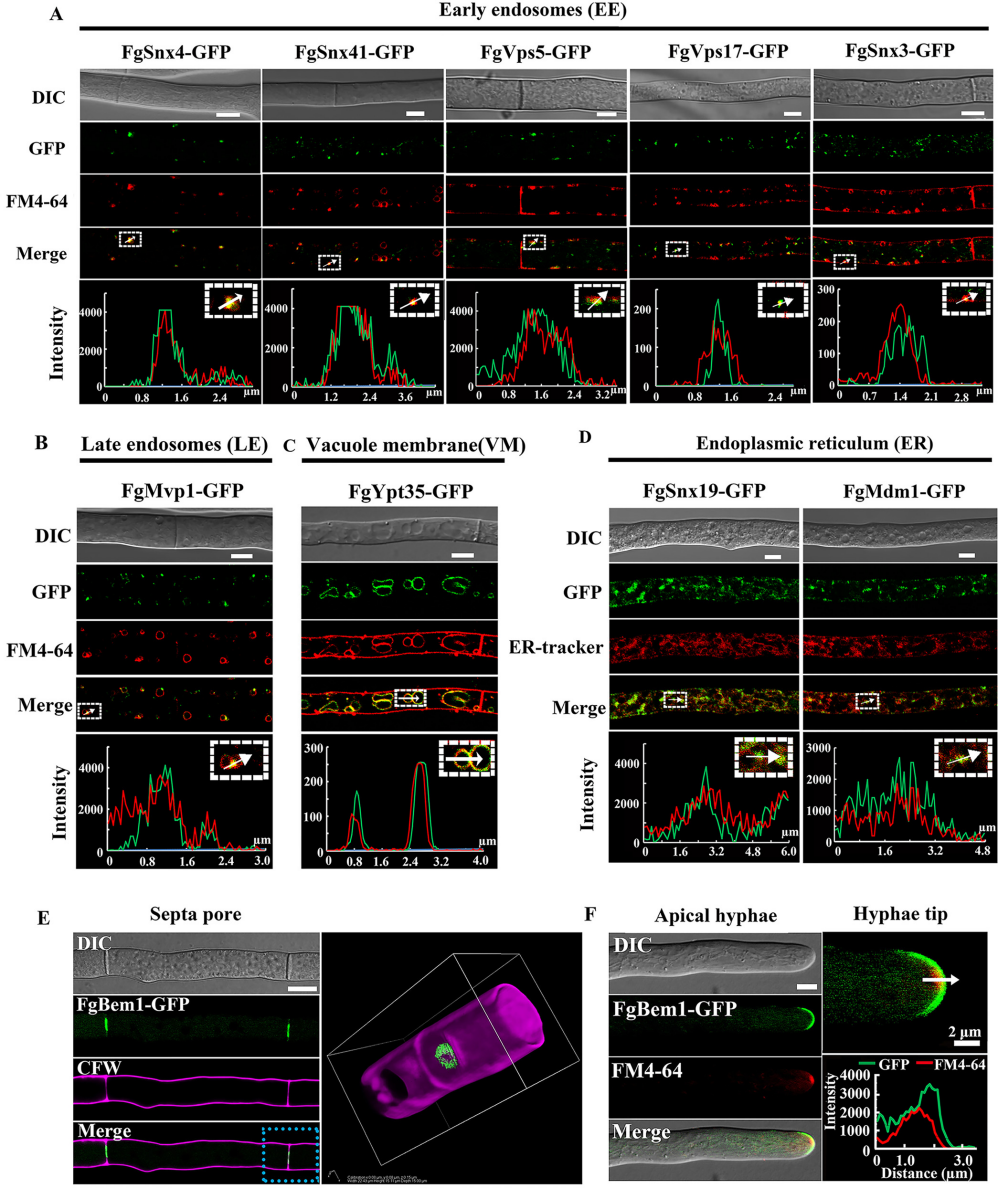
Subcellular localizations of the PX domain-containing protein in mycelia of F. graminearum. (A) Micrographs and their corresponding line scan showing the early endosomal localization of FgSnx3-GFP, FgVps17-GFP, FgVps5-GFP, FgSnx4-GFP, and FgSnx41-GFP in which the GFP signals of the proteins overlap the endocytosis marker FM4-64 in mycelia grown on CM medium. Arrows indicate colocalization. Bars, 5 μm. (B) Punctate FgMvp1-GFP is closely associated with the vacuolar membrane stained with the endocytosis marker FM4-64 in mycelia. Line scan graphs were generated at the indicated positions (arrows) to confirm the late-endosomal localization of FgMvp1-GFP. Bar, 5 μm. (C) Micrographs and the corresponding line scans showing the vacuolar localization of FgYpt35-GFP in which the GFP signal of the protein overlaps with the endocytosis marker FM4-64 in mycelia grown on CM medium. Bar, 5 μm. (D) Micrographs and the corresponding line scan showing the localization of FgSnx19-GFP and FgMdm1-GFP in which the GFP signal of the protein overlaps with the ER marker (ER-tracker) in fresh mycelia grown on CM medium. Bars, 5 μm. (E) Colocalization and three-dimensional reconstruction of FgBem1-GFP (green) and Calcofluor white (purple) staining cell wall and septum. Bar, 5 μm. FgBem1-GFP is concentrated at the septal pore and (or) septal center. (F) Polarized localization of FgBem1-GFP in vegetative hyphae. FgBem1-GFP partially colocalized with the Spitzenkörper (Spk) stained with FM4-64 and is found preferentially at the surface crescent, the region of the plasma membrane immediately in front of the Spk core. Bar, 5 μm. A line scan graph was generated at the indicated position (arrow) to confirm the subtle localization of FgBem1-GFP (green) and Spk (red).

Most of the PX domain-containing proteins in F. graminearum appear as punctate structures in the fungal cells (Fig. S2). We speculate that these punctate structures were either early endosomes (EE) or late endosomes (LE). To verify this, a fluorescent dye, FM4-64, which has been shown to visualize the endosomal structures and vacuolar membrane in filamentous fungi (39), was used to stain the hyphae of the PXs-GFP strains and was visualized by confocal microscopy. As shown in Fig. 1, FgSnx4, FgSnx41, FgVps5 FgVps17, and FgSnx3 localized to the EE, while FgMvp1 and FgYpt35 were observed to colocalize with the LE and vacuolar membrane, respectively (Fig. 1A to C). Since FgSnx19-GFP and FgMdm1-GFP appear as some reticular structures in the cells, we presupposed that these reticular structures were the endoplasmic reticulum (ER). We therefore used ER-tracker as a marker of the ER to stain the hyphae of the FgSnx19-GFP and FgMdm1-GFP transformants grown in CM. The FgSnx19-GFP and FgMdm1-GFP signals consistently colocalized with the ER-tracker (Fig. 1D), suggesting that FgSnx19 and FgMdm1 mainly localize to the ER.

### FgBem1 appears as a disk-like structure around the septal pore and surface crescent of the growing hyphal tips.

Among the 14 PX domain-containing proteins identified in F. graminearum, one, designated FgBem1, was found to have a unique subcellular localization (Fig. S2; Fig. 1E and F). High-resolution laser confocal microscopy, following Calcofluor white (CFW) staining of the hyphae expressing FgBem1-GFP, revealed that the GFP signals of the protein mainly concentrate as a disk-like structure around the septal pores and at the apical hyphae (Fig. 1E and F). To further confirm the apical localization of the FgBem1-GFP, FM4-64 was used to stain the hyphae of the FgBem1-GFP strain. Close examination of individual growing hyphal tip reveals that the FgBem1-GFP mainly localizes to the surface crescent, a region around the plasma membrane and ahead of the Spitzenkörper (Spk) core of the growing hyphal tips. Fluorescence intensity analysis further confirmed the spatial localization between the crescent-shaped FgBem1-GFP and spherical Spk (Fig. 1F; Video S1). These results suggest that FgBem1 participates in the apical extension of the hyphae.

10.1128/mBio.02324-21.8 Video S1 Dynamics of FgBem1-GFP in the growing hyphae tip of F. graminearum. Download Movie S1, AVI file, 1.1 MB. Copyright © 2021 Lou et al. 2021 Lou et al. https://creativecommons.org/licenses/by/4.0/ This content is distributed under the terms of the Creative Commons Attribution 4.0 International license.

It is well-known that microtubules and microtubule-associated proteins form a longer-range transport system for vesicle delivery toward the actin-rich apex (40, 41). The actin cytoskeletons are also essential for endocytosis, which plays a significant role in controlling hyphal morphology (42). We therefore tested whether the localization of FgBem1-GFP is dependent on microtubule and/or actin cytoskeleton. Freshly harvested conidia and hyphae were treated with the actin inhibitor latrunculin A (LatA) and the microtubule-destabilizing agent nocodazole (22). When the growing hyphae were treated with LatA, the hyphal tips appeared as swollen structures (Fig. S3), similar to what is observed when some other filamentous fungi are treated with inhibitors of the actin cytoskeleton (43). Obviously, the surface crescent localization of FgBem1-GFP was replaced by rather diffused cytoplasmic fluorescent signals in the hyphal tips treated with LatA, while the microtubule-destabilizing agent nocodazole has no effect on the apical localization of the FgBem1-GFP (Fig. S3). Noticeably, the septal pore localization of FgBem1-GFP remains unaffected in the fungal hyphae after treatment with either LatA or nocodazole (Fig. S3). Taken together, these data show that disruption of actin and microtubule does not affect the septal localization of FgBem1-GFP, but its hyphal tip localization depends on the integrity of the actin cytoskeleton.

10.1128/mBio.02324-21.3 FIG S3 Actin is required for the localization of FgBem1-GFP to the surface crescent. The localization of FgBem1-GFP was observed after treatment with 10 μm latrunculin A (the actin inhibitor) and 100 μm nocodazole (the microtubule-destabilizing agent), respectively, compared with the DMSO control. Bars, 5 μm. Download FIG S3, TIF file, 2.0 MB. Copyright © 2021 Lou et al. 2021 Lou et al. https://creativecommons.org/licenses/by/4.0/ This content is distributed under the terms of the Creative Commons Attribution 4.0 International license.

### FgBem1 and FgSpo14 are critically important for vegetative growth, conidiation, and sexual development of F. graminearum.

**FIG 2:**
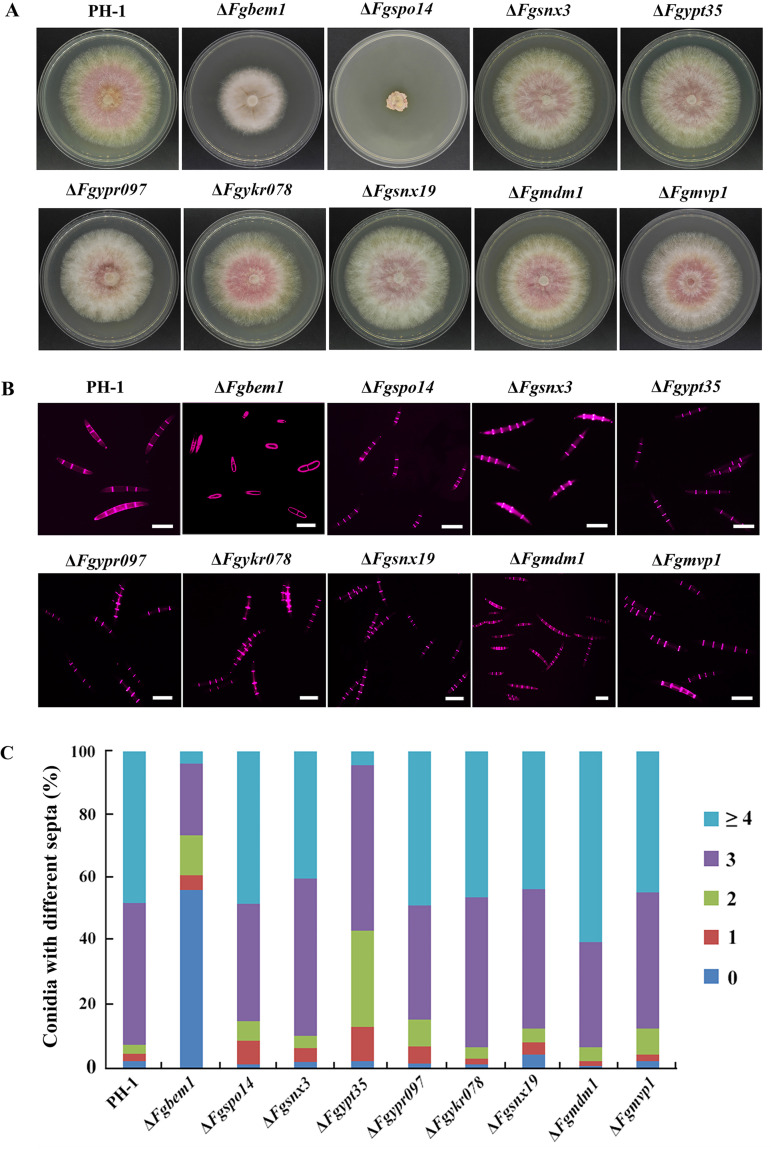
Defects of the PX domain-containing genes deletion mutants in growth and septum formation. (A) Colonies of the wild-type strain PH-1, Δ*Fgbem1*, Δ*Fgspo14*, Δ*Fgsnx3*, Δ*Fgypt35*, Δ*Fgypr097*, Δ*Fgykr078*, Δ*Fgsnx19*, Δ*Fgmdm1*, and Δ*Fgmvp1* mutants grown on CM for 3 days. (B) Conidia from the wild-type strain PH-1 and the gene deletion mutants were stained with Calcofluor white (CFW) to visualize septa under a fluorescence microscope. Bars, 20 μm. (C) Bar chart shows the percentage of spores in each strain containing 0 to 4 septa (*n* > 200) during asexual development.

**FIG 3:**
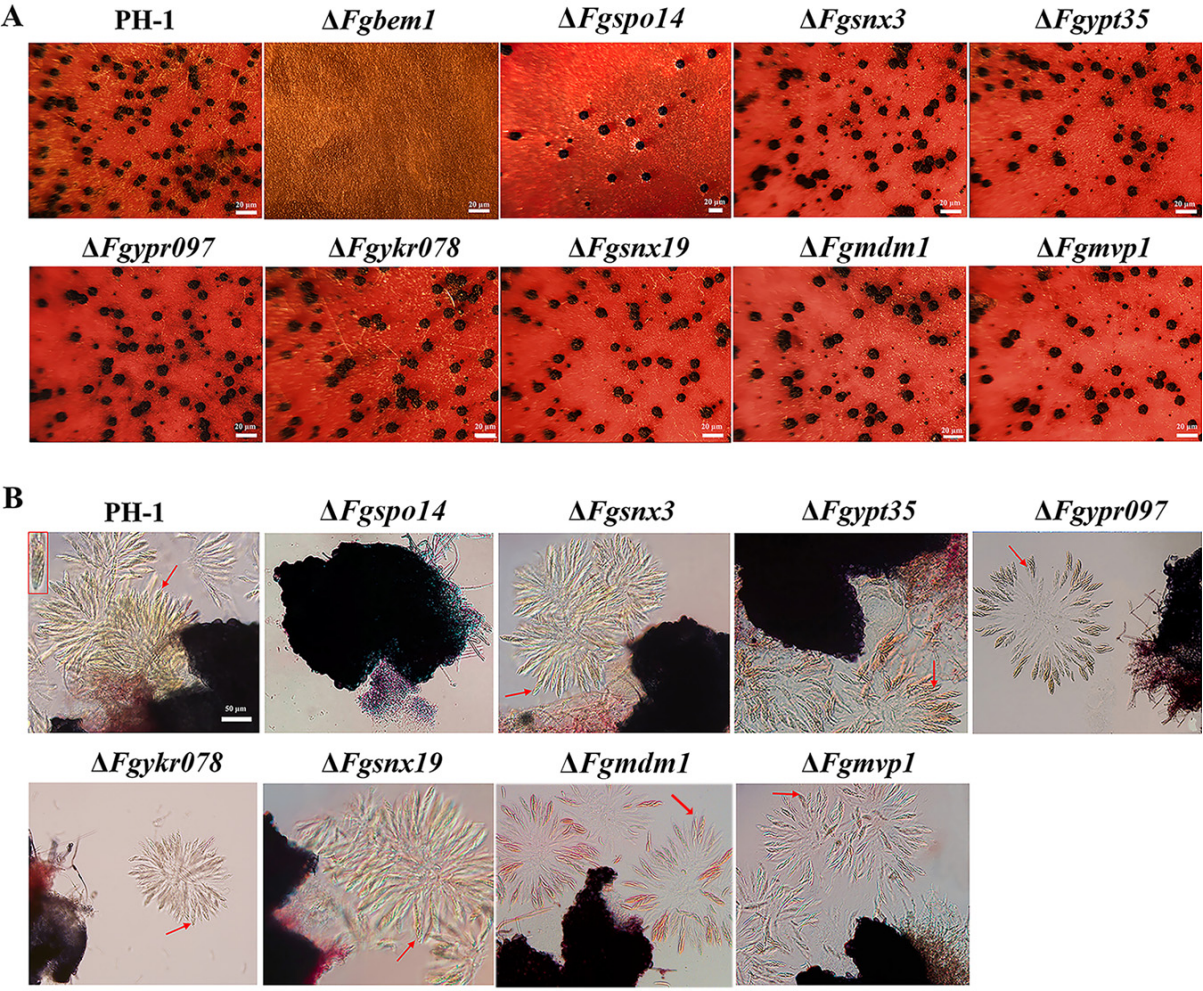
Defects of the PX domain-containing gene deletion mutants in sexual reproduction. (A) Perithecia formation in PH-1 and mutants on carrot agar plates after 14 days. Bars, 20 μm. The formation of perithecia was not observed on carrot agar plates in Δ*Fgbem1* mutants. (B) Fascicles of asci were released from perithecium of the PH-1 and mutants. Bar, 50 μm. Red arrows indicate the asci released from the perithecia of each strain.

To investigate the biological functions of all the PX domain-containing proteins in F. graminearum, we deleted the genes coding for each of the protein products except *FgVPS5*, *FgVPS17*, *FgSNX4*, *FgSNX41*, and *FgVAM7*, which have already been characterized in previous studies (22, 23, 28–30). Each of these genes was replaced by the hygromycin phosphotransferase (*HPH*) gene as a selectable marker using homologous recombination strategy (44). The 9 PX domain-containing gene deletion mutants were confirmed by Southern blotting (Fig. S4), and each strain (including the complemented strains) is listed in Table S1. The wild type and the various deletion mutants with their respective complemented strains were grown on CM solid media for 3 days, after which the growth rates were monitored and compared. The results show that the Δ*Fgbem1* mutant was reduced by 16% in growth rate compared to the PH-1 (Table 2), and it forms colonies with sparse aerial hyphae (Fig. 2A). The Δ*Fgspo14* mutant was reduced by 84% in growth rate, suggesting that the *FgSPO14* gene is critically required for normal vegetative growth of F. graminearum. Notably, the conidiation capacities of all the mutants, except Δ*Fgmvp1* and Δ*Fgykr078*, are obviously reduced compared to that of the PH-1 (Table 2). Interestingly, the number of septa present in each of the Δ*Fgbem1* mutant conidia was reduced compared to the wild-type strain PH-1. While 92.6% of the PH-1 conidia had 3 to 4 septa per conidium, 73.3% of the Δ*Fgbem1* mutant conidia had 0 to 2 septa per conidium (Fig. 2B and C), confirming the critical role of FgBem1 in F. graminearum septation. In addition to conidiation, the ascospores produced by F. graminearum also play a critical role in its infection cycle as primary inoculums (45–47). When grown on carrot agar to induce perithecia and ascospores formation, the wild-type strain PH-1 and most of the PX domain-containing mutants produced abundant perithecia and ascospores after self-fertilization for 14 days (Fig. 3), suggesting that the PX family genes were dispensable for sexual reproduction in F. graminearum. In contrast, the Δ*Fgbem1* mutant completely failed to form any perithecium (Fig. 3). However, very few perithecia of Δ*Fgspo14* mutant form asci and ascospores. These results indicate that FgBem1 and FgSpo14 play a critical role during sexual development.

10.1128/mBio.02324-21.4 FIG S4 Southern blot analyses showing confirmation of targeted gene deletion. (A) Targeted gene deletion of *FgBEM1.* XhoI and KpnI double-digested DNAs showed a 1,650-bp band in PH-1 and 4,059-bp band in the mutants. (B) Targeted gene deletion of *FgSPO14.* EcoRI and KpnI double-digested DNAs showed a 1,793-bp band in PH-1 and 2,770-bp band in mutants. (C) Targeted gene deletion of *FgSNX3.* NcoI-digested DNAs showed a 2,142-bp band in PH-1 and 1,833-bp band in the mutants. (D) Targeted gene deletion of *FgYPT35.* KpnI digested DNAs showed a 2,117-bp band in PH-1 and 4,155-bp band in the mutants. (E) Targeted gene deletion of *FgYPR097.* ClaI-digested DNAs showed a 5,329-bp band in PH-1 and 7,260-bp band in the mutants. (F) Targeted gene deletion of *FgYKR078.* HindIII-digested DNAs showed a 1,705-bp band in PH-1 and 2,846-bp band in the mutants. Δ*FgYKR078*-1 was verified as a negative transformant. (G) Targeted gene deletion of *FgSNX19.* XhoI-digested DNAs showed a 2,829-bp band in PH-1 and 5,477-bp band in the mutants. (H) Targeted gene deletion of *FgMDM1.* HindIII-digested DNAs showed a 2,000-bp band in PH-1 and 3,527-bp band in the mutants. (I) Targeted gene deletion of *FgMVP1.* ClaI-digested DNAs showed a 3,261-bp band in PH-1 and 4,070-bp band in mutants. Download FIG S4, TIF file, 1.0 MB. Copyright © 2021 Lou et al. 2021 Lou et al. https://creativecommons.org/licenses/by/4.0/ This content is distributed under the terms of the Creative Commons Attribution 4.0 International license.

### The PX domain-containing proteins play different roles in response to hyperosmotic stress, cell wall-damaging agents, and/or oxidative stress in F. graminearum.

Membrane vesicles production plays an important role in increasing pathogen adaptabilities in the presence of stress-inducing factors (48). To explore the roles of the PX domain-containing proteins in the stress resistance in F. graminearum, we inoculated the wild type and these gene deletion mutants on CM media containing 1.5 M KCl (ionic and osmotic stress inducer), 0.7 M NaCl (ionic and osmotic stress inducer), 1 M d-sorbitol (osmotic stress inducer), 10 mM H_2_O_2_ (oxidative stress inducer), 0.02% SDS (cell wall-damaging agents), 200 μg/ml CFW (cell wall-damaging agents), and 3 mg/ml Congo red (CR) (cell wall-damaging agents), respectively. The colony diameters were analyzed after 3 days, and then the relative growth inhibition rates (RGI) were measured (Fig. S5). Compared to the wild type, the Δ*Fgspo14* showed significantly reduced sensitivity to 1 M KCl and 0.7 M NaCl, but growth of the other mutants was suppressed to various extents from 1.1% to 7.5%. Among these, Δ*Fgypt35*, Δ*Fgypr097*, and Δ*Fgmdm1* showed enhanced sensitivity to 1 M KCl. Furthermore, all the other mutants, except for Δ*Fgsnx19*, were enhanced for sensitivity to 0.7 M NaCl, suggesting different roles of the PX domain proteins in hyperosmotic/ionic stress tolerance. Additionally, Δ*Fgspo14*, Δ*Fgypt35*, Δ*Fgsnx19*, and Δ*Fgmdm1* mutants showed significantly increased sensitivities to 1 M d-sorbitol. Moreover, all the PX domain gene deletion mutants, with the exception of Δ*Fgykr078*, showed reduced sensitivities to the cell wall-damaging agents CR and CFW, indicating the important roles of these PX domain proteins in the cell wall integrity maintenance. However, Δ*Fgspo14* and Δ*Fgbem1* mutants were slightly reduced in the sensitivities to 0.02% SDS. Interestingly, we found that all the PX domain deletion mutants, except for Δ*Fgbem1*, showed reduced sensitivity to oxidative stress induced by 10 mM H_2_O_2._ Nevertheless, the RGI of Δ*Fgbem1* was increased to 100% compared with that of PH-1 (Fig. S5). Taken together, these results indicate that the PX domain-containing proteins play different roles in response to hyperosmotic stress, cell wall-damaging agents, and/or oxidative stress in F. graminearum.

10.1128/mBio.02324-21.5 FIG S5 Sensitivity of the wild-type strain PH-1 and all PX domain family genes deletion mutants to osmotic stresses, oxidative stress, and cell wall-damaging agents. (A) Colonies of PH-1 and all gene deletion mutants on CM media supplemented with 1 M KCl, 0.7 M NaCl, 1 M d-sorbitol, 10 mM H_2_O_2_, 0.02% (wt/vol) SDS, 200 μg/ml CFW, and 1 mg/ml CR, respectively. Colony diameters (B) and mycelial radial growth inhibition rates (C) were quantified after incubation on CM media supplemented with different stress-inducing agents after 3 days postinoculation. Red asterisks indicate statistically significant difference (*P* < 0.05). Download FIG S5, TIF file, 2.8 MB. Copyright © 2021 Lou et al. 2021 Lou et al. https://creativecommons.org/licenses/by/4.0/ This content is distributed under the terms of the Creative Commons Attribution 4.0 International license.

### All the PX domain-containing proteins, except FgMvp1 and FgYkr078, are important for virulence and DON production in F. graminearum.

**FIG 4:**
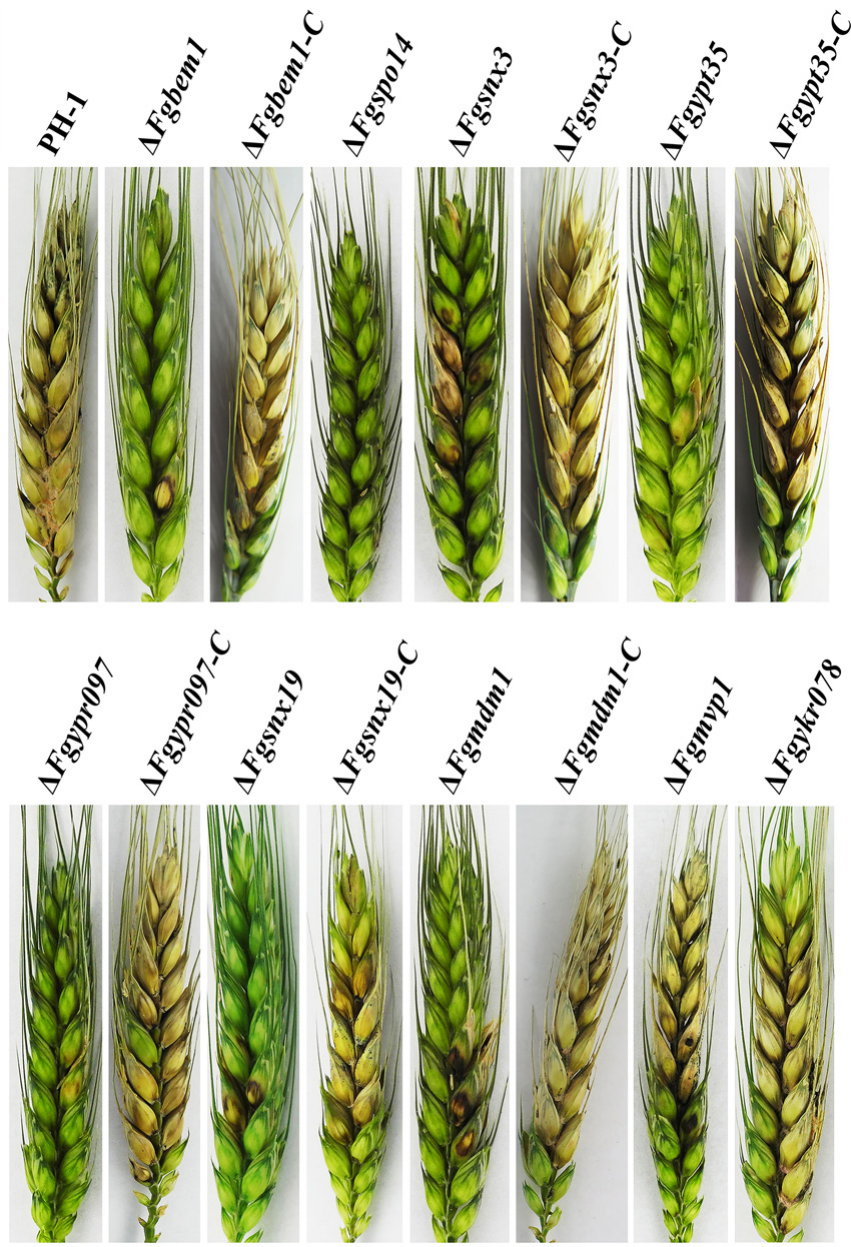
Infection of flowering wheat heads with the PX domain-containing gene mutants. Flowering wheat heads were inoculated with conidia from the wild type (PH-1), Δ*Fgbem1*, Δ*Fgbem1-*C, Δ*Fgspo14*, Δ*Fgsnx3*, Δ*Fgsnx3-*C, Δ*Fgypt35*, Δ*Fgypt35-*C, Δ*Fgypr097*, Δ*Fgypr097-*C, Δ*Fgsnx19*, Δ*Fgsnx19*-C, Δ*Fgmdm1*, Δ*Fgmdm1*-C, Δ*Fgmvp1*, and Δ*Fgykr078*. The brown lesions were observed on the flowering wheat heads following infection with the PH-1 and the mutants. Three independent experiments were conducted.

In F. graminearum, loss of FgVps5 and FgVps17, which served as retromer complex subunits in yeast, causes significant defects in development and virulence (22). Further study showed that the endosomal recycling of FgSnc1 (a SNARE protein) mediated by FgSnx41-FgSnx4 heterodimer is essential for polarized growth and pathogenicity of F. graminearum (23). These results suggest that PX domain-containing proteins may play a critical role in the virulence of F. graminearum. To further verify this hypothesis, we carried out the infection assays for the various strains in the present study. As a positive control, the wild-type strain showed severe disease symptoms and had an average disease index (number of diseased spikelets per head) of approximately 13.2. However, seven of the mutants, including Δ*Fgbem1*, Δ*Fgspo14*, Δ*Fgsnx3*, Δ*Fgypt35*, Δ*Fgsnx19*, Δ*Fgypr097*, and Δ*Fgmdm1*, had severe defects in virulence, with average disease indexes of 1.8, 1.2, 7.1, 4.2, 3.6, 2.2, and 3.2, respectively (Fig. 4; Table 2). Considering the fact that DON is one of the best-characterized virulence factors in F. graminearum (49), we further quantified and compared the level of DON produced by the various strains. Compared to the wild type, the levels of DON produced by all of the seven PX domain-containing gene deletion mutants, except Δ*Fgmvp1* and Δ*Fgykr078*, are significantly reduced in TBI medium (Table 2), which is consistent with their defects in plant infection. Since Δ*Fgbem1*, Δ*Fgspo14*, Δ*Fgsnx3*, Δ*Fgypt35*, Δ*Fgsnx19*, Δ*Fgypr097*, and Δ*Fgmdm1* showed different degrees of defects in the pathogenicity, we performed gene complementation by reintroducing each gene along with its native promoter and succeeded in generating all the complemented strains except for Δ*Fgspo14*, due to its extremely slow growth and consequent difficulty to obtain a sufficient number of protoplasts for transformation experiments. The DON production and disease indexes of all the complemented strains (Δ*Fgbem1-C*, Δ*Fgsnx3-C*, Δ*Fgypt35-C*, Δ*Fgsnx19-C*, Δ*Fgypr097-C*, and Δ*Fgmdm1-C*) were restored to the levels observed in the PH-1 (Fig. 4; Table 2). Taken together, these results suggested that all PX domain-containing proteins, except FgMvp1 and FgYkr078, are important for virulence and DON production in F. graminearum.

### FgBem1 targets septal pores and is retained in the septum pores after actin constriction during the septum development.

**FIG 5:**
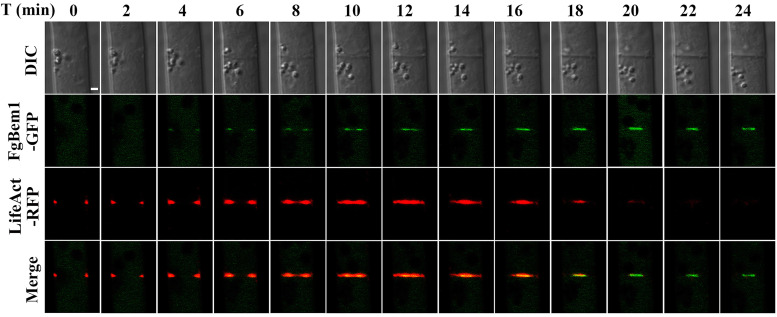
Septal development in F. graminearum hyphae was visualized by dynamic tracing of FgBem1-GFP and LifeAct-RFP. FgBem1-GFP starts to show up after formation of LifeAct-RFP-marked contractile ring, while it accumulated at the septum center after F-actin depolymerization. Bar, 2 μm.

The results presented earlier suggest that FgBem1 plays a critical role in fungal septation. To further validate this assertion, we visualized the dynamic localization of FgBem1-GFP and LifeAct-red fluorescent protein (RFP) during the process of septa formation. Because LifeAct-RFP binds specifically to F-actin, it has been widely used as a marker for tracing the constricting ring during septation processes among filamentous fungi (50). Consistent with previous studies, the LifeAct-RFP fluorescence emerged very early (0 min) at future septation sites and then moved from the periphery to the center of the ring between 0 and 18 min and disappeared after about 20 min, leaving only a few patches occasionally (Fig. 5; Video S2). FgBem1-GFP started to appear at the same time with LifeAct-RFP at the septation site from 4 min and continued to coexist until after 18 min when the LifeAct-RFP signal diminished. At the maturity time of the septa (20 min), LifeAct-RFP was absent (Fig. 5; Video S2), whereas FgBem1-GFP was still associated with the septa and formed a structure at the center of the mature septa (>20 min) (Fig. 5; Video S2). These results suggested that FgBem1 targets to the septal pore, where it is accompanied by actin constriction and may play important roles in the late stages of septation during the septal development.

10.1128/mBio.02324-21.9 Video S2 Comotility of FgBem1-GFP and LifeAct-RFP during the septum development in F. graminearum. Download Movie S2, AVI file, 3.4 MB. Copyright © 2021 Lou et al. 2021 Lou et al. https://creativecommons.org/licenses/by/4.0/ This content is distributed under the terms of the Creative Commons Attribution 4.0 International license.

### FgBem1 is necessary for normal nuclear division in F. graminearum.

**FIG 6:**
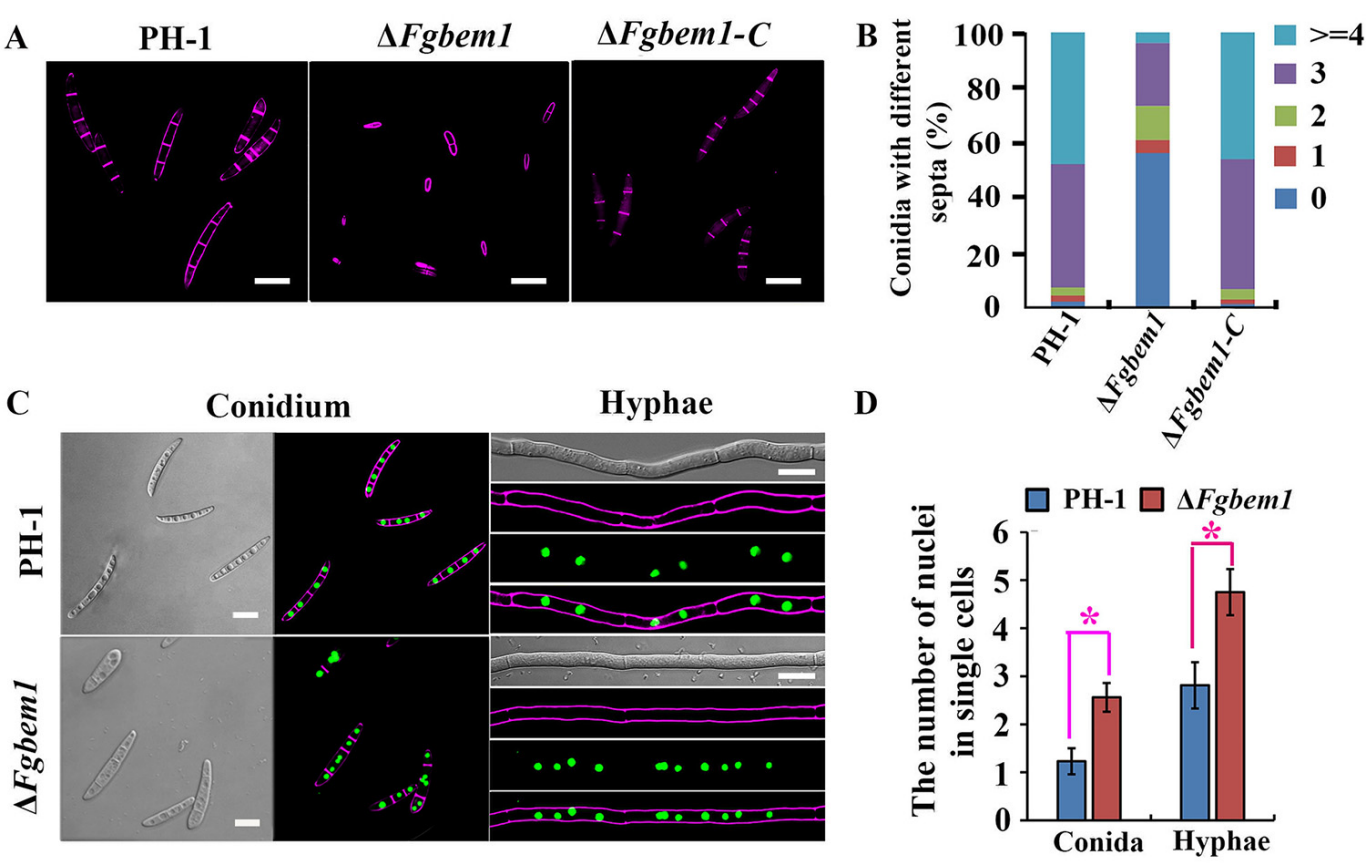
Defects of Δ*Fgbem1* mutant in nucleus division. (A) Conidia of the PH-1, Δ*Fgbem1*, and Δ*Fgbem1*-C were stained with CFW. Bars, 20 μm. (B) The number of septa in the conidia of the PH-1, Δ*Fgbem1*, and Δ*Fgbem1*-C. (C) The hyphae of the PH-1 and Δ*Fgbem1* expressing the H1-GFP construct were stained with CFW. Bars, 10 μm. (D) The average number of nuclei in single-conidial and hyphal cells (*n* > 300) of PH-1 and Δ*Fgbem1* expressing the H1-GFP construct. Red asterisks indicate statistically significant difference (*P* < 0.05).

Since FgBem1 plays a critical role in septation, we supposed that the defect in septum formation of the Δ*Fgbem1* mutant may, in turn, cause a defect in nuclear division. A histone-GFP (H1-GFP) fusion construct was transformed into the protoplasts of PH-1 and Δ*Fgbem1* mutant, respectively. As expected, GFP signals were observed in the nuclei of both conidia and hyphae of the H1-GFP transformants from the PH-1 and Δ*Fgbem1* (Fig. 6). The conidia harvested from the carboxymethylcellulose (CMC) culture and fresh hyphae were then stained with CFW and examined by fluorescence microscopy. As shown in Fig. 6A and B, the average number of nuclei per cell in a conidium of the Δ*Fgbem1* mutant is 2.6, while that of the PH-1 is 1.3 per cell. We also examined the distribution of nuclei in the fungal hyphae and observed that most (92%) of the nuclei in the wild-type strain PH-1 were distributed evenly within the hyphae, and the average number of nuclei per hyphal cell was 2.5 (Fig. 6C and D). In contrast, the average number of nuclei per hyphal cell of the Δ*Fgbem1* mutant was 4.6 (Fig. 6C and D). These results demonstrate the important role of FgBem1 in regulating normal nuclear division in F. graminearum.

### FgBem1 is dispensable for polarized secretion but is required for ROS generation during hyphal apical growth.

**FIG 7:**
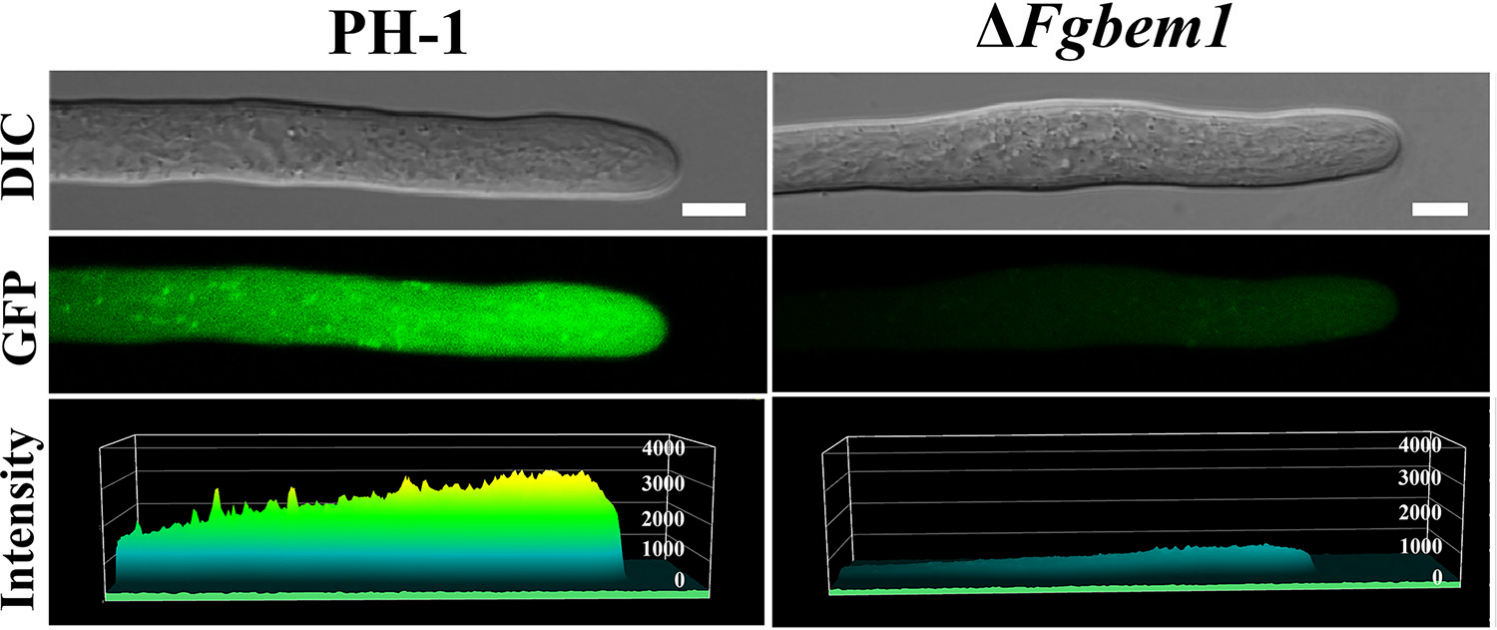
ROS quantification in the hyphae tips of the PH-1 and Δ*Fgbem1* strains using DCFH-DA fluorescence probe. Bars, 5 μm.

A previous study showed that FgSnc1 is a vesicle SNARE protein that accumulates at the active growing site and plays an important role in vesicular polarized secretion in F. graminearum (23). DnfA and DnfB are P-type ATPases, which have been considered cargoes for endocytic collar and are involved in polar growth and asexual sporulation in the filamentous fungus A. nidulans (51). In addition, the organization of the exocyst complex proteins Exo70 and Exo84 at the appressorium pore is a septin-dependent process, which also requires regulated synthesis of ROS by the NoxR-dependent Nox2-NADPH oxidase complex in M. oryzae (52). To determine if FgBem1 is required for vesicular polarized secretion, we introduced FgSnc1-GFP, FgDnfA-GFP, FgDnfB-GFP, and FgExo84-GFP into the PH-1 and Δ*Fgbem1* strains and determined their localizations by confocal microscopy. We found that the localizations of FgSnc1-GFP, FgDnfA-GFP, FgDnfB-GFP, and FgExo84-GFP in the Δ*Fgbem1* mutant are similar to those observed in the PH-1 (Fig. S6), suggesting that FgBem1 is not involved in polarized secretion. Since the endogenous ROS plays a critical role in DON biosynthesis, development, and virulence in F. graminearum (53) and deletion of *FgBEM1* abolished these processes, we decided to dissect the relationship between FgBem1 and ROS production. To achieve this, the vegetative hyphae of the PH-1 and Δ*Fgbem1* mutants were stained with DCFH-DA (2′,7′-dichlorodihydrofluorescein diacetate) fluorescence probe and observed by confocal microscopy. DCFH-DA has been used as a chemical reporter for quantifying the intracellular ROS level in previous studies (54, 55). Our results showed that the level of ROS in the *Fgbem1* mutants was significantly reduced compared to that of the PH-1 (Fig. 7), which supports that FgBem1 plays a critical role in endogenous ROS production during apical extension of the hyphae. These results are also consistent with the earlier finding that the Δ*Fgbem1* mutant is defective in growth on CM supplemented with 10 mM H_2_O_2_. Therefore, we conclude that FgBem1 is dispensable for polarized secretion but required for ROS generation during hyphal apical extension.

10.1128/mBio.02324-21.6 FIG S6 Localization of Snc1-GFP, EXO84-GFP, DnfA-GFP, and DnfB-GFP in the apical hyphae and hyphal septa of PH-1 and Δ*Fgbem1* strains. Bar, 5 μm. Download FIG S6, TIF file, 2.0 MB. Copyright © 2021 Lou et al. 2021 Lou et al. https://creativecommons.org/licenses/by/4.0/ This content is distributed under the terms of the Creative Commons Attribution 4.0 International license.

### Apical targeting of FgBem1 depends on its SH3 domains, while septum anchorage relies on its PX domain.

**FIG 8:**
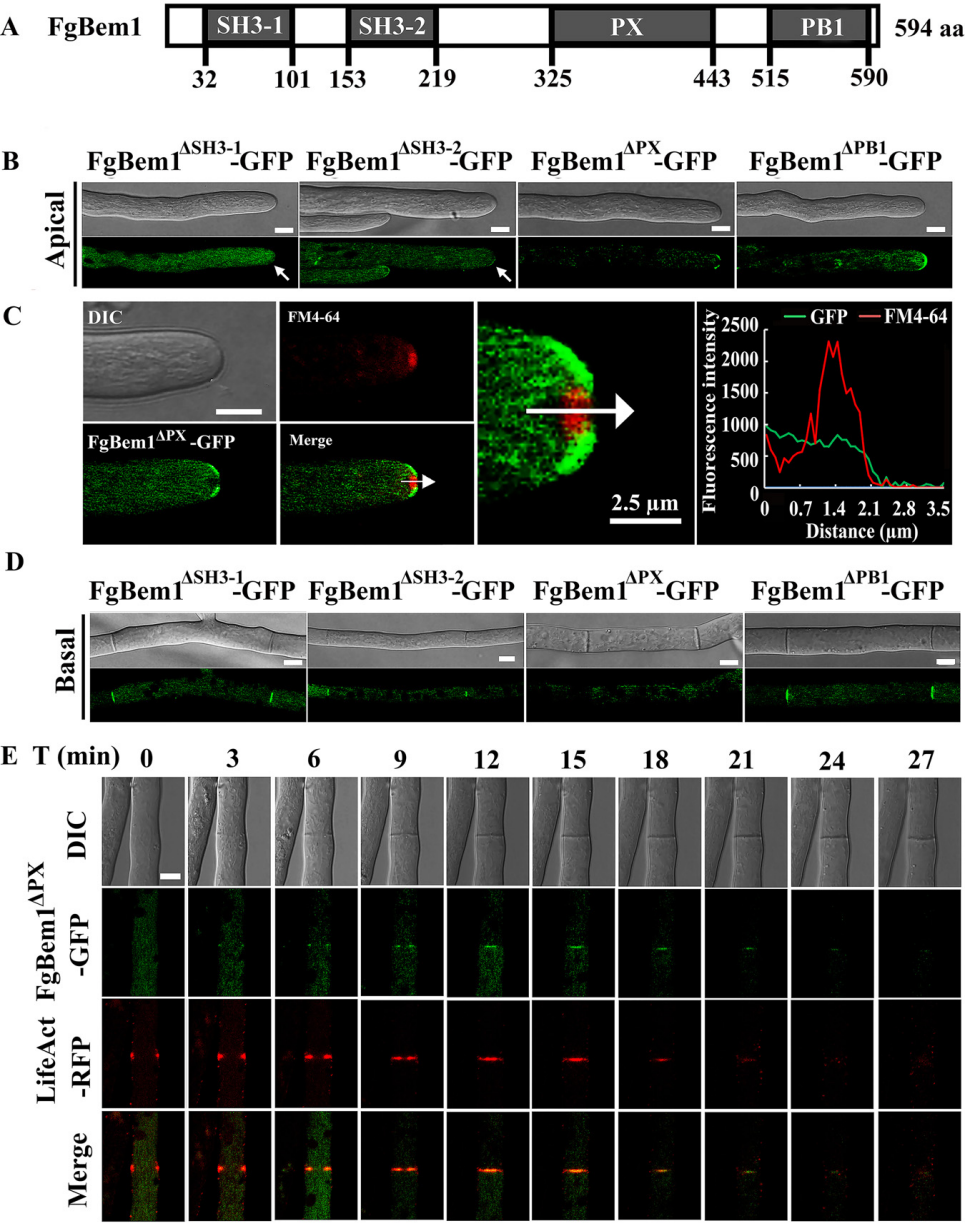
Subcellular localizations of the FgBem1 truncated proteins lacking the various domains in F. graminearum. (A) Schematic diagram of the FgBem1 protein with SH3-1, SH3-2, PX, and PB1 domains highlighted. (B) Apical localization of the truncated proteins in hyphal tips of Δ*Fgbem1* mutants. Deletion of either SH3-1 or SH3-2 domain leads to detectable depolarization of FgBem1. Bars, 5 μm. (C) The PX domain is essential for proper polar localization of FgBem1 protein. FgBem1^ΔPX^-GFP failed to colocalize with the Spitzenkörper (Spk) at the hyphal tips of F. graminearum stained with FM4-64. Bar, 5 μm. The line scan graph was generated at the position shown by the arrow, which characterizes the different locations of FgBem1^ΔPX^-GFP (green) and Spk (red) at the hyphal tip. (D) Localization of FgBem1^ΔSH3-1^-GFP, FgBem1^ΔSH3-2^-GFP, FgBem1^ΔPX^-GFP, and FgBem1^ΔPB1^-GFP at the septal pore. Bars, 5 μm. (E) The PX domain is essential for stabilizing the localization of FgBem1-GFP to the septal center. Septal development in F. graminearum hyphae was visualized by dynamic tracing of FgBem1^ΔPX^-GFP and LifeAct-RFP. Bar, 2 μm.

To further investigate the roles of each domain of the FgBem1 in the subcellular localization and biology functions, we introduced four truncated forms of the FgBem1-GFP, designated FgBem1^ΔSH3-1^-GFP, FgBem1^ΔSH3-2^-GFP, FgBem1^ΔPX^-GFP, and FgBem1^ΔPB1^-GFP, individually into protoplasts of the Δ*Fgbem1* mutant (Fig. 8A). Compared to the FgBem1^ΔPX^-GFP and FgBem1^ΔPB1^-GFP, the truncated FgBem1^ΔSH3-1^ and FgBem1^ΔSH3-2^ proteins had undetectable surface crescent localization of FgBem1, suggesting that SH3-1 and SH3-2 domains are essential for the proper apical targeting of the FgBem1 protein (Fig. 8B). Interestingly, although the FgBem1^ΔPX^-GFP was still able to localize to the apex sites of the growing tips, its vertex signals were lost on the surface crescent, which was further confirmed by comparison of FgBem1^ΔPX^-GFP and Spk in the growing hyphal tip (Fig. 8C). In addition, we analyzed the localization of four truncated forms of the FgBem1-GFP on septum. The results show that loss of the PX domain in FgBem1 leads to an undetectable or unstable GFP signal at matured septa in mycelium (Fig. 8D). Using time-lapse series imaging, we further confirmed that FgBem1^ΔPX^-GFP disappears after the formation of the matured septa, demonstrating that the PX domain is essential for the stability of FgBem1 at the septal pore (Fig. 8E; Video S3). These results revealed that apical targeting of FgBem1 depends on its SH3 domains, while septum anchorage relies on its PX domain.

10.1128/mBio.02324-21.10 Video S3 Comotility of FgBem1^ΔPX^-GFP and LifeAct-RFP during septum development in F. graminearum. Download Movie S3, AVI file, 2.4 MB. Copyright © 2021 Lou et al. 2021 Lou et al. https://creativecommons.org/licenses/by/4.0/ This content is distributed under the terms of the Creative Commons Attribution 4.0 International license.

### The SH3, PX, and PB1 domains of FgBem1 are required for sexual development and virulence in F. graminearum.

**FIG 9:**
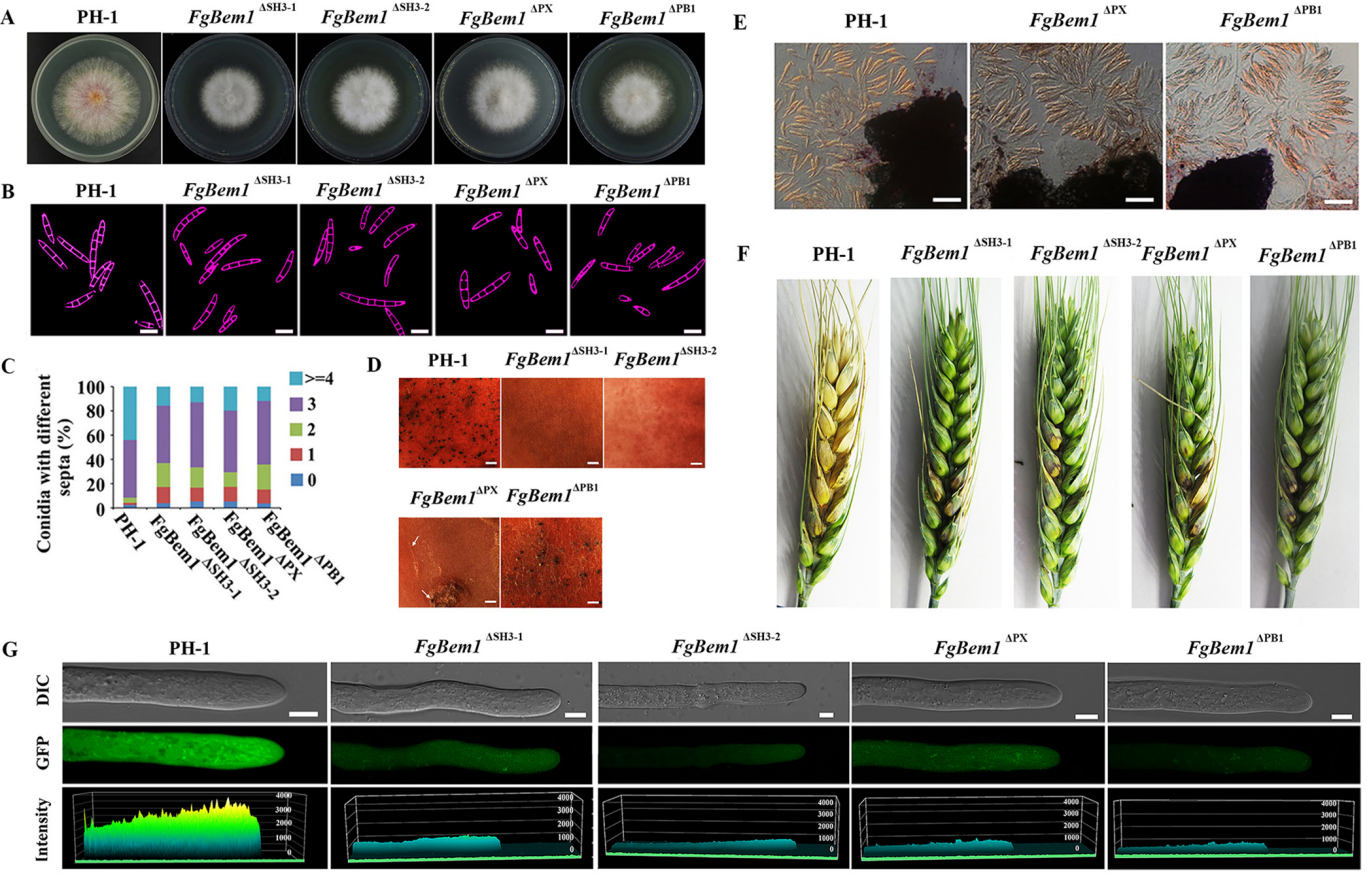
Phenotypes of the FgBem1 domain-truncated mutants in F. graminearum. (A) Colonies of the wild-type (PH-1) and FgBem1 domain-truncated mutants grown on complete medium (CM) after 3 days. (B) Conidia of the PH-1 and FgBem1 domain-truncated mutants were stained with Calcofluor white to visualize septa under a fluorescence microscope. Bars, 20 μm. (C) Number of septa in the conidia of the PH-1 and FgBem1 domain-truncated mutants. (D) Perithecium formation in the PH-1 and FgBem1 domain-truncated mutants on carrot agar plates after 20 days. Bars, 10 μm. (E) Ascospores released from the perithecia of the PH-1 and FgBem1 domain-truncated mutants. Bars, 50 μm. (F) Wheat coleoptiles were inoculated with mycelial plugs of the PH-1 and FgBem1 domain-truncated mutants. The brown lesions observed on the coleoptiles (in length and discoloration intensity) were distinguishable in PH-1 compared to the FgBem1 domain-truncated mutants. (G) ROS accumulation level was observed using DCFH-DA fluorescence probe in hyphae tip of the PH-1 and the FgBem1 domain-truncated mutants. Bars, 5 μm.

Next, we determined the functions of the four domains of FgBem1 in fungal development and virulence. Compared to PH-1, all the four truncated mutants of FgBem1 showed reduction in vegetative growth and conidiation (Fig. 9A; Table 2). To further investigate the septal development of the four domain-truncated mutants, the conidia harvested from CMC culture of all four mutants after incubation for 4 days were stained with CFW. Noticeably, the defect of Δ*Fgbem1* mutant in septum formation was partially rescued in each of the four domain-truncated mutants (FgBem1^ΔSH3-1^, FgBem1^ΔSH3-2^, FgBem1^ΔPX^, and FgBem1^ΔPB1^) (Fig. 9B and C). In terms of sexual reproduction, FgBem1^ΔPX^ and FgBem1^ΔPB1^ mutants were slightly rescued in the sexual reproduction defect observed in Δ*Fgbem1* mutant (small perithecia and normal ascospore) compared to FgBem1^ΔSH3-1^ and FgBem1^ΔSH3-1^ mutants, which still failed to form perithecia (Fig. 9D and E). Compared to the wild-type strain, the four domain-truncated mutants were significantly defective in virulence (Fig. 9F). It was also notable that the ROS generation in the four domain-truncated mutants was still significantly reduced compared to that of the PH-1 (Fig. 9G). These results reveal that the four domains of FgBem1 are required for growth, conidiation, sexual reproduction, septation, ROS production, and virulence in F. graminearum.

## DISCUSSION

PX domain-containing proteins are poorly studied in filamentous fungi, especially in plant-pathogenic fungi. In this study, we systematically identified and characterized the functions of PX domain-containing proteins in F. graminearum and established their subcellular localizations in normal growth and toxin-induced conditions. To our knowledge, this is the first detailed and comprehensive functional analysis of PX domain-containing proteins in fungi. In addition, we deeply analyzed one PX domain-containing protein, FgBem1, in septation-associated nucleus division and ROS generation during hyphal apical growth, which are important for fungal development and pathogenesis. These findings also provide new insights into PX domain-involved septal development in filamentous fungi.

In general, the PX domain consists of a sequence of 100 to 140 amino acid residues conserved from yeast to human (9). Our phylogenetic analysis revealed that there are 14 PX domain-containing proteins in the genome of F. graminearum, while 49 PX domain-containing proteins exist in mammals, and those are roughly grouped into two categories, 34 candidate SNXs and 15 other PX domain-containing proteins (10). Increasing evidence supports that SNXs are endosome-localized proteins and are involved in vesicle trafficking (56). The endocytic network comprises a series of interconnected tubulovesicular membranous compartments that regulate various sorting and signaling events (57). In the present study, the systemic subcellular localization analysis reveals that 9 PX domain-containing proteins are localized to endomembranes, such as early or late endosomes (FgSnx3, FgMvp1, FgVps5, FgVps17, FgSnx4, and FgSnx41), vacuolar membrane (FgYpt35), and endoplasmic reticulum (FgSnx19, FgMdm1). This is consistent with the conserved functions of these proteins in membrane trafficking. Further genetic study (plus previously reported studies) showed that the mutants of these proteins are severely defective in DON production (a virulence factor) in F. graminearum (Table 1) (49, 58, 59), which implies that there may be a potential link between endosomal trafficking and DON production/secretion. We especially found that FgSnx3, FgVps5, and FgVps17 localized to early endosomes (Fig. 1A), which is consistent with the colocalization of the homolog GRD19 and retromer complex on the tubular endosomes in S. cerevisiae (60). Our findings revealed that FgYpt35 specifically localized to the vacuolar membrane (Fig. 1C), which is consistent with the vacuolar rim localization of Ypt35 as a novel adaptor binding with Vps13 in S. cerevisiae (61). Interestingly, we observed, for the first time, that the FgSnx19-GFP and FgMdm1-GFP proteins are localized to some distinct patches in the endoplasmic reticulum in F. graminearum (Fig. 1D), which may be anchored in the ER as a novel interorganelle tether, similar to what is seen in yeast (19). It is worth investigating the roles of these protein-mediated interorganelle communication networks in further study and uncovering their potential molecular mechanism in fungal pathogenicity.

In addition to the endomembrane localization of most of PX domain-containing proteins, we also found that three PX domain-containing proteins, FgSpo14, FgYpr097, and FgYkr078, localize to the cytoplasm. FgSpo14 was previously annotated as FgPLD1 since it contains two phospholipase D (PLD) domains except for the N-terminal PX domain (62). Functional analysis of FgPLD1 revealed that it is important for hyphal growth, conidiation, and plant infection, which is consistent with our study (62). However, our data showed that loss of *FgSPO14* does not completely abolish perithecia formation, which is a bit different from the previous report of FgPLD1. We speculate that the difference could have arisen due to the use of different background strains and incubation periods.

Most interestingly, our data showed that FgBem1 specially localizes to hyphal septal pores and the surface crescent of the growing hyphal tips (Fig. 1E and F). Deletion of *FgBEM1* resulted in defect in fungal polarize growth. In S. cerevisiae, Bem1 is involved in symmetry breaking and polarization during bud formation, where it appears to constitute a parallel mechanism to actin-based cell polarization (63). In A. nidulans and E. festucae, homologues of Bem1 protein localize to the growing hyphal tips, which rhymes with their conserved role as a polarity factor. However, the function disruption of these Bem1 homologues had only minor consequences on general hyphal polarity in both species (36, 64). In addition, the Bem1 homologue of E. festucae (BemA) was found to interact with NoxR, a regulatory component of NADPH oxidases involved in ROS production (36). In the present study, we found that FgBem1 also localizes to growing hyphal tips, but loss of FgBem1 function had a significant negative effect on the fungal polarized growth (Fig. 1 and Table 1), which is different from that observed in N. crassa (38). Interestingly, the Δ*Fgbem1* mutant lost the ability to survive on CM media supplemented with 10 mM H_2_O_2_ (Fig. S5), and the ROS accumulation level in the mutants was significantly reduced (Fig. 7), suggesting that FgBem1 may be a potential shared ancestral link between fungal polarity establishment and ROS generation in F. graminearum. Recently, ROS has been recognized as a key player in the complex signaling network of plants’ stress responses (65–68). Thus, it remains possible that the defect of Δ*Fgbem1* mutant in virulence may be either due to the mutant’s growth defect or its failure to overcome plant defense responses. In N. crassa, the PX domain is dispensable for the localization and function of Bem1, and the two SH3 domains have partially redundant functions during cell communication and directional growth (38). However, in our study, we found that the two SH3 domains and PX domain are both essential for hyphal tip localization of FgBem1 and also crucial for fungal vegetative growth, sporulation, and sexual reproduction (Table 1; Fig. 8 and 9).

In filamentous fungi, septum formation occurs with certain regularity, indicative of a rather close correlation between mitosis and septum formation, which may be indispensable during sexual development, vegetative growth, and pathogenesis. In F. graminearum, only a few septa were found in asexual spores, vegetative hyphae, and ascospores of the septin mutants, which caused defects in pathogenicity (69). FgCdc14, a member of phosphatases, was demonstrated to be required for nuclear division and septa formation. The *FgCDC14* deletion mutant was defective in ascosporogenesis and pathogenesis in F. graminearum (70). FgRho4, as a core component of Rho GTPases, was shown to be involved in nuclear division and septum formation and is required for hyphal growth and conidiation (71). In this study, we found that septation in *FgBEM1* deletion mutant was reduced, and hyphae or conidia compartments of the mutant contained more nuclei than that of PH-1, indicating defects in coordination between nuclear division and cytokinesis. It is also interesting to test a possible connection between FgBem1 and FgRho4 or FgCdc14 in septation. The process of septation gives the fungus the ability to undergo differentiation specifically in conidiation or sporulation and formation of tissue-like structures (50). A previous study provides an important clue to sustaining the hypothesis that a close correlation exists between the septation and ascospore formation, which may be indispensable for the pathogenesis of F. graminearum (71). In the present study, ascospore formation was disrupted in Δ*Fgbem1* mutants. In addition, Δ*Fgbem1* showed severe defects in the pathogenesis of F. graminearum (Fig. 3 and 4). These results suggest that septation may be required for effective sexual reproduction, which is, in turn, very important for the pathogenicity of F. graminearum. Intriguingly, we found, for the first time, that the PX domain-truncated protein of FgBem1 localizes around the septal pores in a ringlike structure and subsequently disappeared along with actin patches after septation was completed (Fig. 8E), suggesting that the anchorage of the FgBem1 protein to the septal pore is likely to be mediated by the PX domain.

## MATERIALS AND METHODS

### Strains and culture conditions.

The wild-type strain (PH-1) and all the PX domain-containing gene deletion mutants of F. graminearum generated in this study (supporting information given in Table S1A in the supplemental material) were routinely cultured at 25°C on CM (6 g yeast extract, 6 g casein hydrolysate, and 10 g sucrose in 1 liter double-distilled water [ddH_2_O]). Growth rates on CM plates and conidiation in liquid carboxymethylcellulose (CMC) medium were evaluated as described previously (72). For sensitivity tests to the various stress-inducing agents, 0.7 M NaCl, 1 M KCl, 1 M d-sorbitol, 10 mM H_2_O_2_, 0.02% SDS, 200 μg/ml Calcofluor white (CFW), and 1 mg/ml Congo red (CR), respectively, were prepared in CM agar plates (73). For sexual reproduction, perithecia formation was assayed on carrot agar medium as previously described (74). All experiments were repeated at least three times.

### Generation of gene deletion mutants and complementation transformants.

Protoplast preparation and fungal transformation were performed as described previously (44). To delete a target gene, two fragments upstream and downstream of the target gene were amplified using specific primers listed in Table S1C. We then generated the target gene replacement constructs by split-marker approach (75). Subsequently, the resulting constructs carrying the hygromycin split marker were transformed into the wild-type strain PH-1. The resulting transformants were first screened by PCR with two designated primer pairs as shown in Table S3 and further characterized by Southern blotting using the digoxigenin High Prime DNA labeling and detection starter kit 1 (Roche, Mannheim, Germany). To generate the GFP fusion construct (native promoter::ORF::GFP), the entire PX domain-containing gene sequence, including its native promoter and its open reading frame, was amplified with the primers shown in Table S1C. Subsequently, these sequences were seamlessly cloned to the KpnI/HindIII-digested *pKNTG* plasmid by using ClonExpress II one-step cloning kit (Vazyme Biotech Co., Ltd.), respectively. All resulting plasmids were verified by sequencing analysis and are listed in Table S1B. The resulting constructs were then transformed into the protoplasts of the deletion mutants, respectively. Neomycin-resistant transformants were screened by PCR with OF/SGFP-R (Table S1C) and confirmed by examining the signals of green fluorescent protein (GFP) using epifluorescence microscopy. At least two positive transformants were used for phenotypic analyses.

### Generation of FgBem1^ΔSH3-1^-GFP, FgBem1^ΔSH3-2^-GFP, FgBem1^ΔPX^-GFP, and FgBem1^ΔPB1^-GFP fusion constructs.

To generate the FgBem1^ΔPX^-GFP fusion construct in which the PX domain was deleted, we first used PCR to amplify the FgBem1^ΔPX^-GFP-1 and FgBem1^ΔPX^-GFP-2 fragments from genomic DNA of the wild-type strain PH-1 using the primer pairs FgBEM1-CF/FgBEM1-CR-NPX and FgBEM1-CPX-F/FgBEM1-CR. Then, FgBem1^ΔPX^-GFP-1 and FgBem1^ΔPX^-GFP-2 were seamlessly cloned to the KpnI*/*HindIII-digested pKNTG by using ClonExpress II one-step cloning kit, designated FgBem1^ΔPX^-GFP. The resulting plasmid FgBem1^ΔPX^-GFP was verified by sequencing analysis. FgBem1^ΔSH3-1^-GFP, FgBem1^ΔSH3-2^-GFP, and FgBem1^ΔPB1^-GFP fusion constructs were also generated using a similar approach. All the primers used are listed in Table S1C. All the resulting *FgBEM1*-truncated constructs were transformed into the Δ*Fgbem1* mutant to assay the functions of each domain.

### Plant infection and DON production assays.

Infection assays on flowering wheat heads and wheat coleoptiles were conducted as previously described (4). For DON production assays, the conidia harvested from CMC cultures with a final concentration of 1 × 10^5^ conidia/ml were inoculated into liquid trichothecene biosynthesis induction (TBI) media as described previously (76). Cultures were incubated in the dark without shaking at 28°C for 7 days and then filtrated using miracloth (Merck Millipore, catalogue number: 475855). The filtrate was used for DON production measurement using an enzyme-linked immunosorbent assay (ELISA)-based DON quantification kit (Beacon Analytical Systems, Saco, ME, USA) according to the manufacturer’s instructions. All experiments were repeated three times.

### Cytological analyses and time-lapse microscopy.

For hyphal branch assays, the “inverted agar block method” of preparing and staining samples was used as described previously (77). FM4-64 (Invitrogen) was used for staining Spitzenkörper, plasma membrane, early endosome, and late endosome at a final concentration of 2 μM (39). To investigate the colocalization of FgBem1-GFP and FM4-64 in the apical hyphae, the FgBem1-GFP-expressing strain was grown on CM agar for 2 days, and then a block of CM agar containing the colony margin was excised and placed on a coverslip with the fungal culture resting directly on the cover slip for confocal microscopic observation. To examine the effects of FgBem1 gene deletion on nuclear division, the PH-1 and Δ*Fgbem1* expressing the H1-GFP construct were grown on CM agar for 2 days and stained with CFW. To examine the effects of the microtubule-destabilizing agent nocodazole and the actin inhibitor latrunculin A (LatA) on the localization of FgBem1-GFP in apical hyphae, FgBem1-GFP-expressing strain was grown on CM agar for 2 days, and then a block of CM agar containing the colony margin was excised and placed directly on a coverslip, which was treated with dimethyl sulfoxide (DMSO) (as control), nocodazole (at a final concentration of 100 μM), or LatA (at a final concentration of 10 μM), respectively. After incubation for 30 min, the samples were observed under a Nikon A1 confocal microscope. For time-lapse measurement of septum development, FgBem1-GFP- and LifeAct-RFP-expressing strains were grown on CM agar plates for 2 days. Using the same strategy described above, FgBem1-GFP and LifeAct-RFP signals were observed using the Nikon A1 confocal microscope at 488 nm and/or 561 nm for 24 min. Images were taken every 2 min. The elapsed times are indicated in the videos. To analyze the effects of FgBem1 deletion on ROS accumulation, the PH-1 and *Fgbem1* mutants were grown on solid CM for 2 days, and then the vegetative hyphae were stained with DCFH-DA fluorescent probe and observed under a Nikon A1 confocal microscope.
