# Supplementary figures and images for: Genome-Wide Characterization of PX Domain-Containing Proteins Involved in Membrane Trafficking-Dependent Growth and Pathogenicity of Fusarium graminearum
Source: mBio. 2021 Dec 21;12(6):e02324-21. doi: 10.1128/mBio.02324-21 (PMC8689521; doi:10.1128/mBio.02324-21)

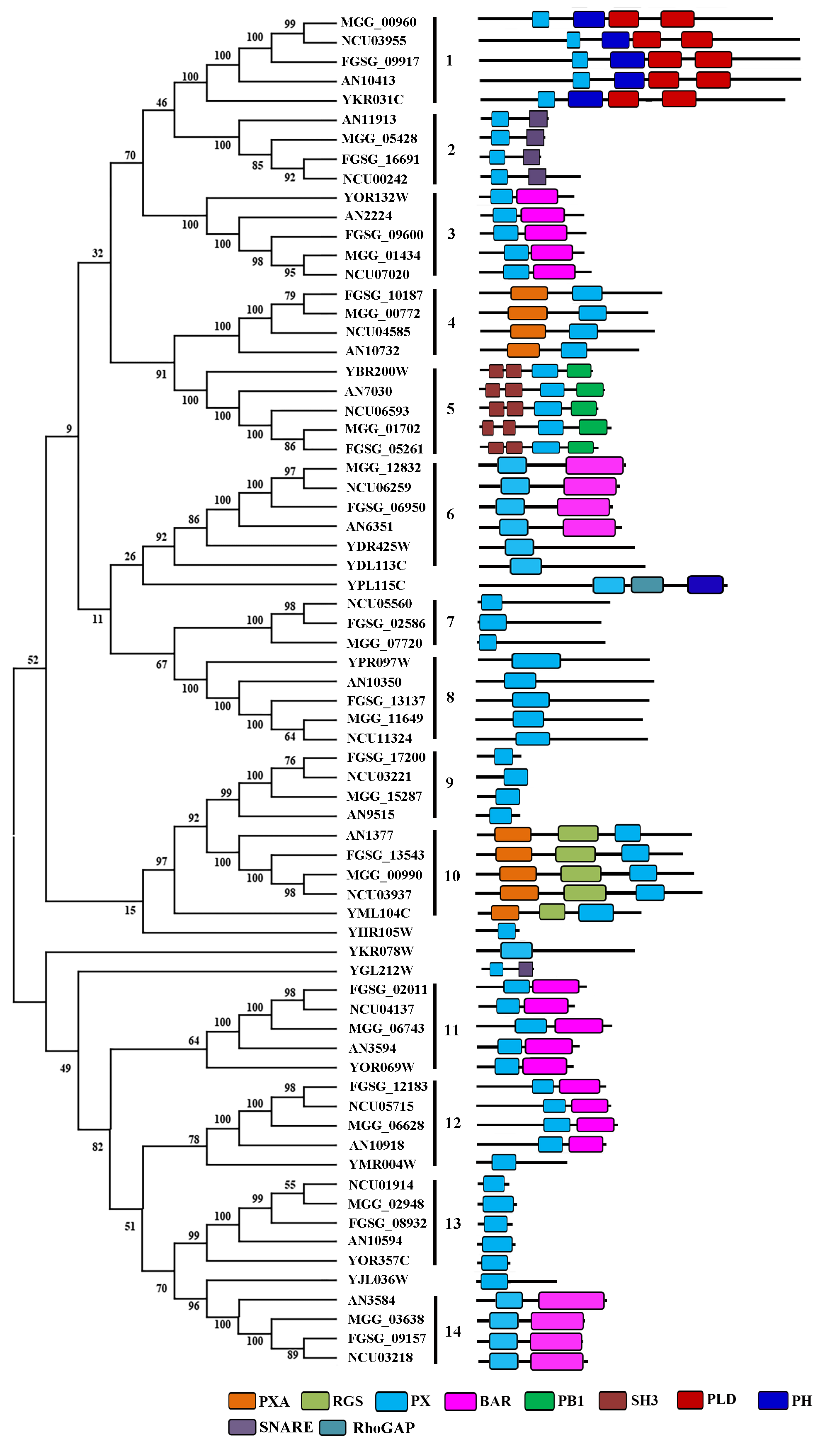

Supplement: FIG S1 [file mbio.02324-21-sf001.tif]

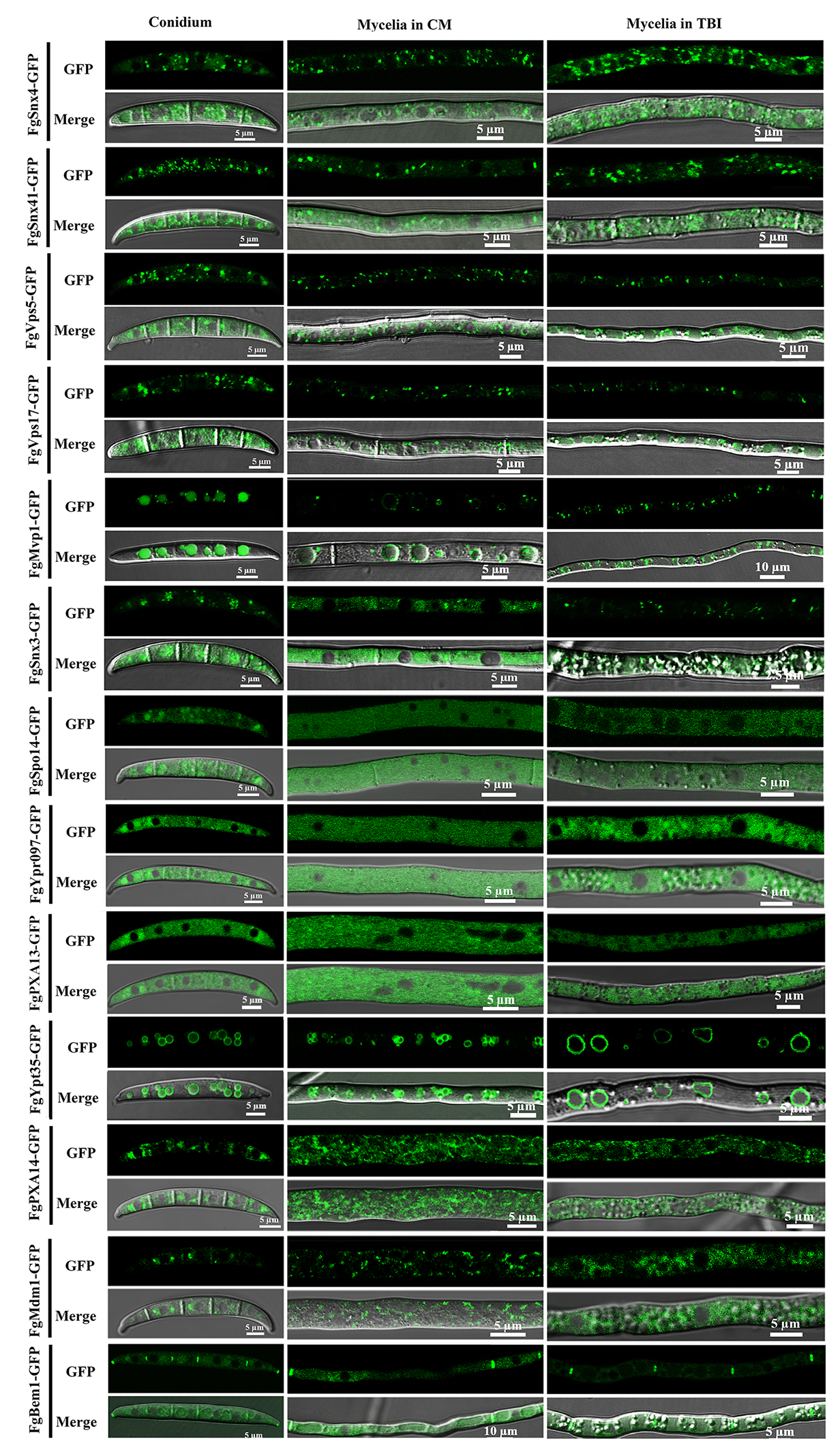

Supplement: FIG S2 [file mbio.02324-21-sf002.tif]

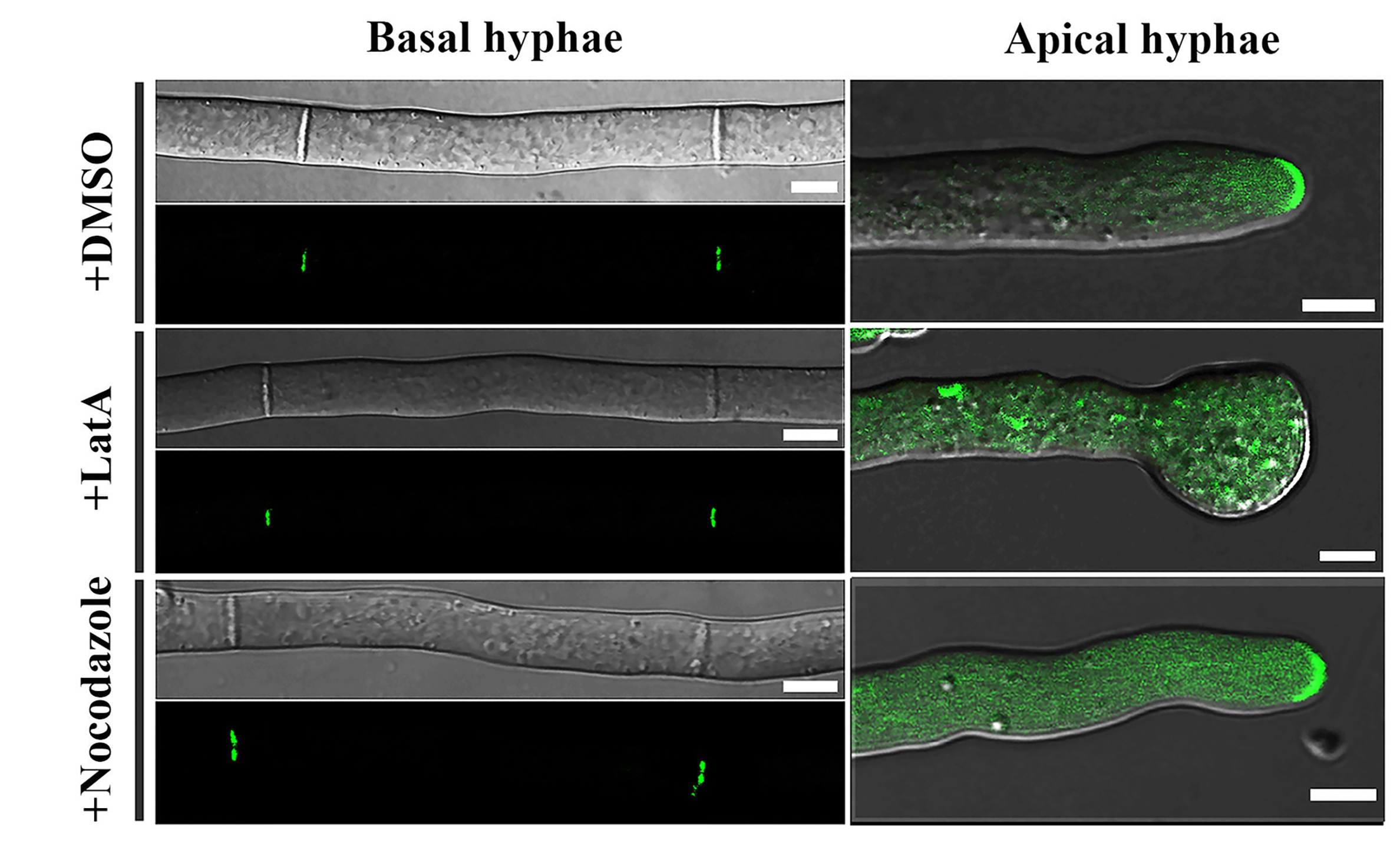

Supplement: FIG S3 [file mbio.02324-21-sf003.tif]

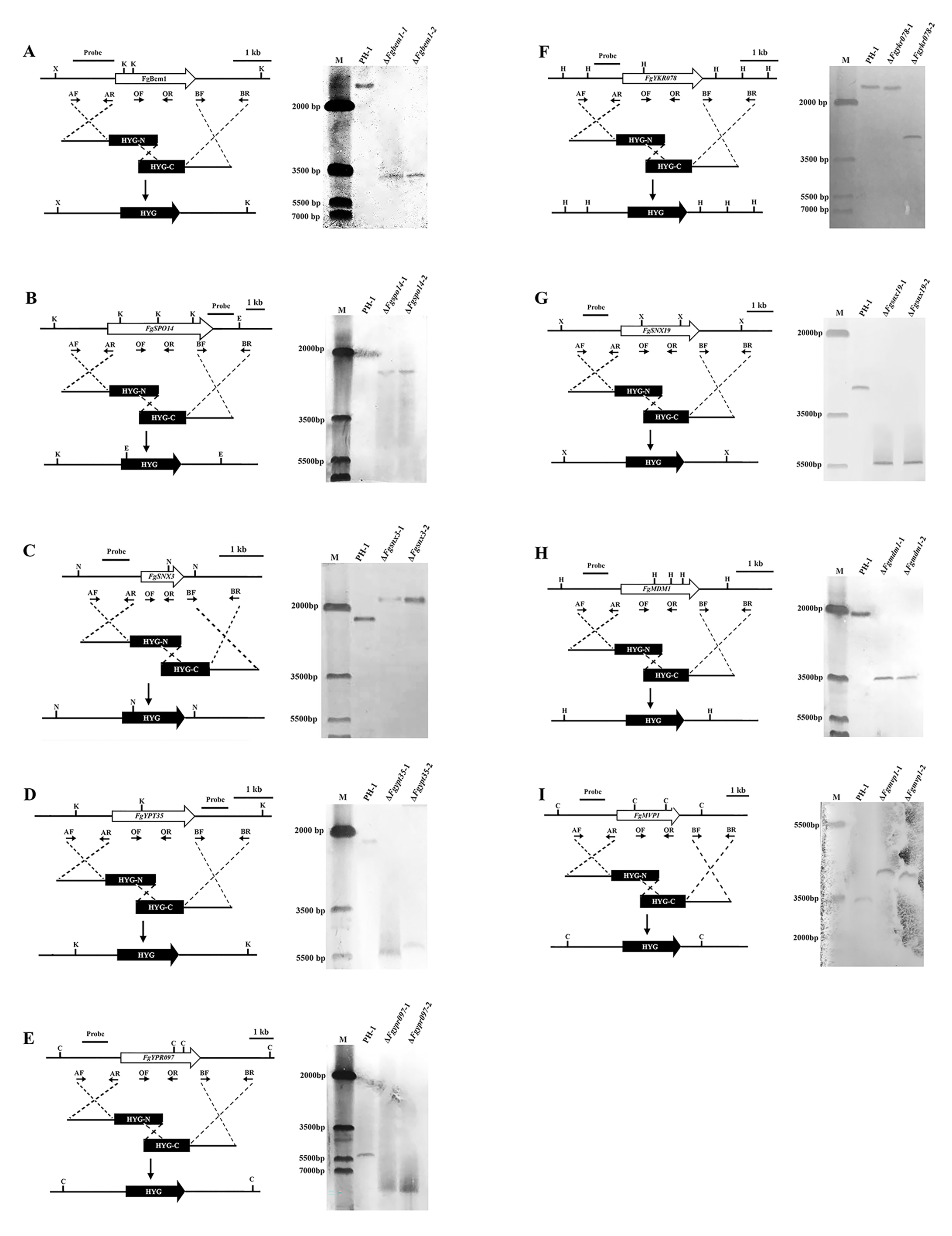

Supplement: FIG S4 [file mbio.02324-21-sf004.tif]

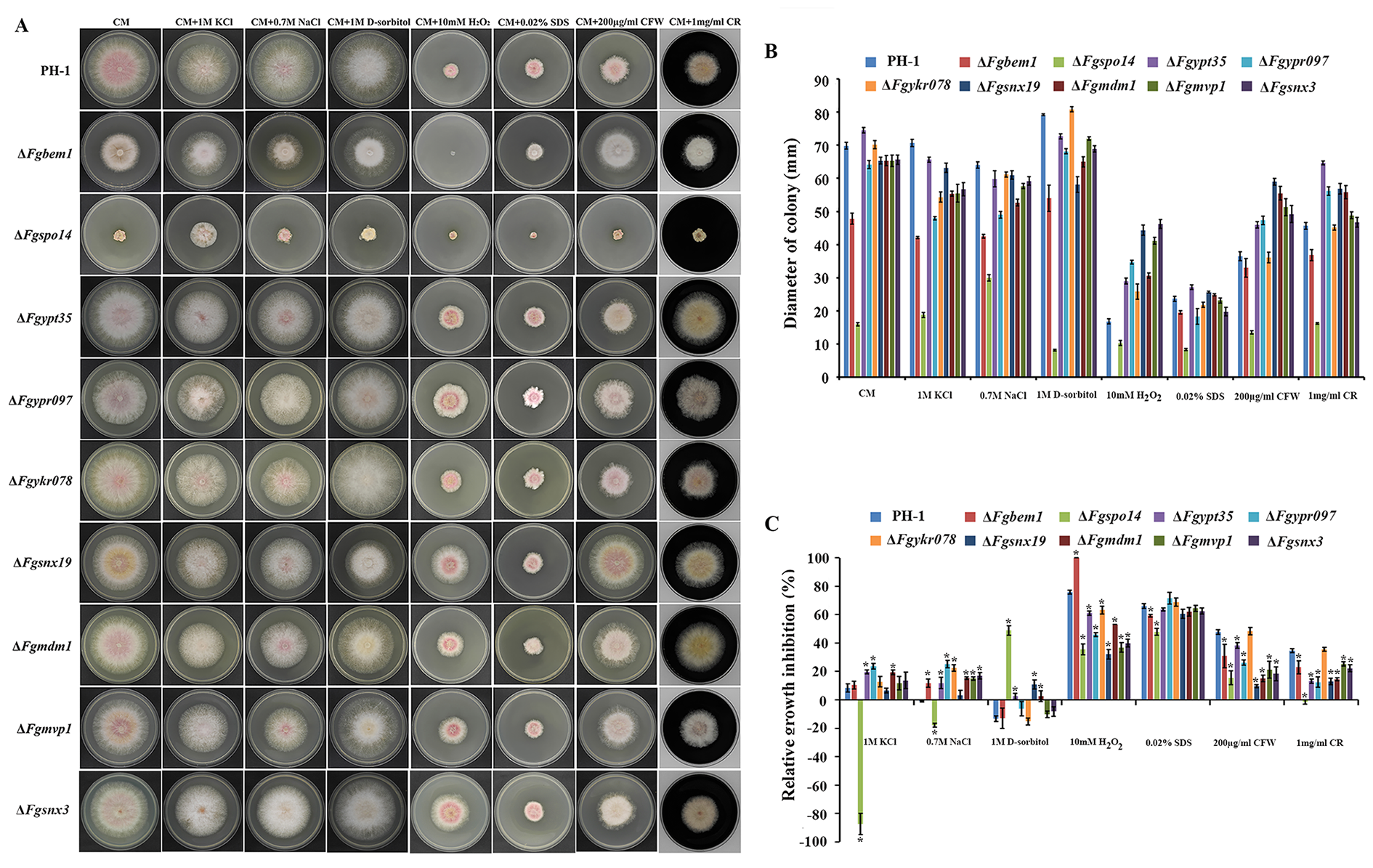

Supplement: FIG S5 [file mbio.02324-21-sf005.tif]

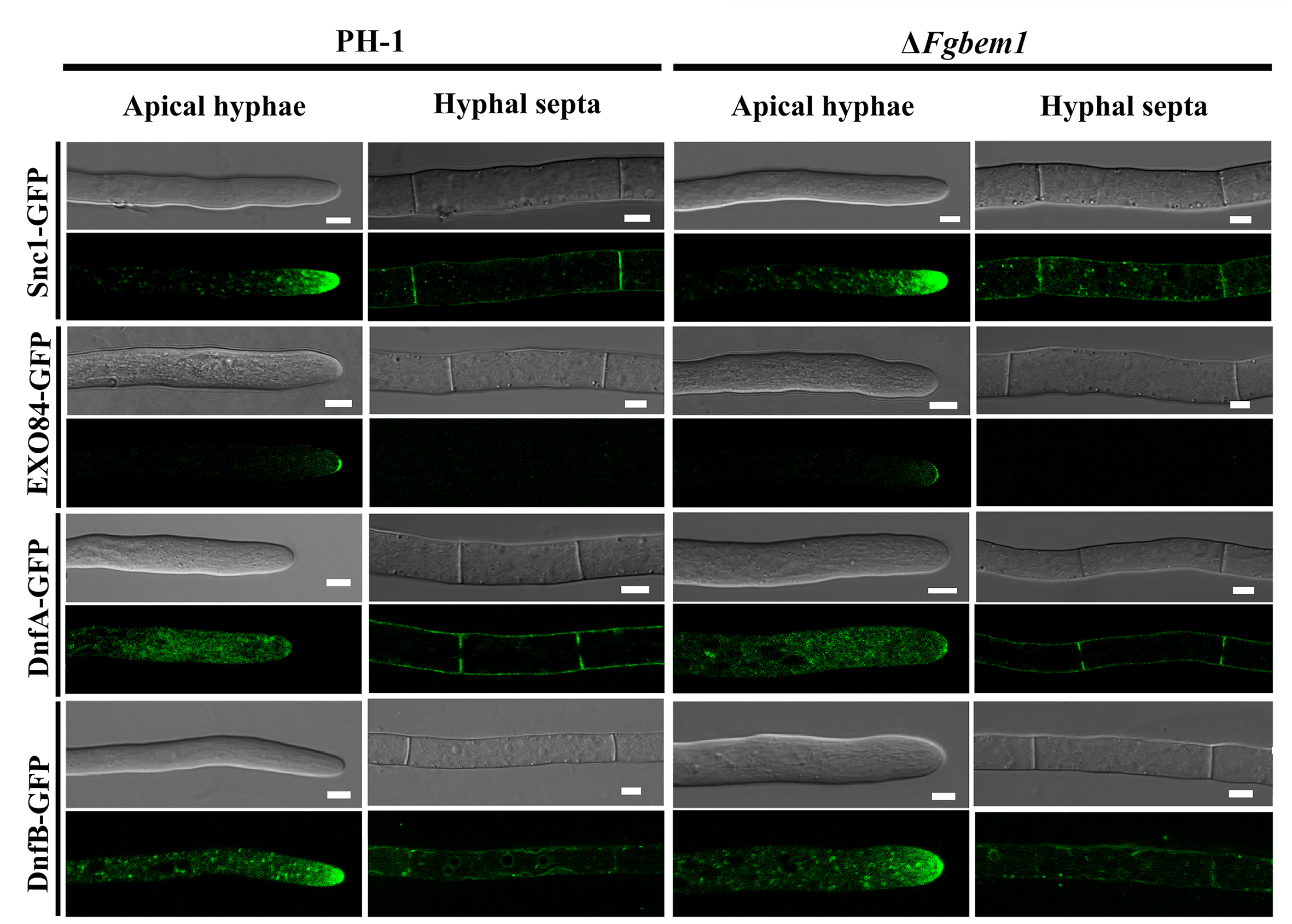

Supplement: FIG S6 [file mbio.02324-21-sf006.tif]
