# Supplementary material for: Genome-Wide Characterization of PX Domain-Containing Proteins Involved in Membrane Trafficking-Dependent Growth and Pathogenicity of Fusarium graminearum
Source: mBio. 2021 Dec 21;12(6):e02324-21. doi: 10.1128/mBio.02324-21 (PMC8689521; doi:10.1128/mBio.02324-21)
Supplement: TABLE S1 [file mbio.02324-21-st001.docx]

**Table S1 The strains, plasmids and PCR primers used in this study**

| **A. The wild-type and mutant strains of fungi used in this study** | | |
| --- | --- | --- |
| **Strain** | **Genotype description** | **Reference** |
| PH-1 | Wild-type | Cuomo *et al*. (2007) |
| Δ*Fgbem1* | *FgBem1* deletion mutant of PH-1 | This study |
| Δ*Fgbem1-C* | Δ*Fgbem1* transformant expressing FgBem1-GFP  construct | This study |
| *FgBem1*ΔSH3-1 | Δ*Fgbem1* transformant expressing  *FgBem1*ΔSH3-1-GFP construct | This study |
| *FgBem1*ΔSH3-2 | Δ*Fgbem1* transformant expressing  *FgBem1*ΔSH3-2-GFP construct | This study |
| *FgBem1*ΔPX | Δ*Fgbem1* transformant expressing  *FgBem1*ΔPX-GFP construct | This study |

| *FgBem1*ΔPB1 | Δ*Fgbem1* transformant expressing  *FgBem1*ΔPB1-GFP construct | | This study |
| --- | --- | --- | --- |
| Δ*Fgspo14* | *FgSPO14* deletion mutant of PH-1 | | This study |
| PH-1+FgSpo14-GFP | PH-1strain expressing FgSpo14-GFP construct | | This study |
| Δ*Fgsnx3* | *FgSNX3* deletion mutant of PH-1 | | This study |
| Δ*Fgsnx3-C* | Δ*Fgsnx3* transformant expressing FgSnx3-GFP  construct | | This study |
| Δ*Fgypt35* | *FgYPT35* deletion mutant of PH-1 | | This study |
| Δ*Fgypt35-C* | Δ*Fgypt35* transformant expressing FgYpt35-GFP construct | | This study |
| Δ*Fgypr097* | *FgYPR097* deletion mutant of PH-1 | | This study |
| Δ*Fgypr097-C* | Δ*Fgypr097* transformant expressing FgYpr097-GFP construct | | This study |
| Δ*Fgykr078* | *FgYKR078* deletion mutant of PH-1 | | This study |
| Δ*Fgykr078-C* | Δ*Fgykr078* transformant expressing FgYkr078-GFP construct | | This study |
| Δ*Fgsnx19* | *FgSNX19* deletion mutant of PH-1 | | This study |
| Δ*Fgsnx19-C* | Δ*Fgsnx19* transformant expressing FgSnx19-GFP  construct | | This study |
| Δ*Fgmdm1* | *FgMDM1* deletion mutant of PH-1 | | This study |
| Δ*Fgmdm1-C* | Δ*Fgmdm1* transformant expressing FgMdm1-GFP  construct | | This study |
| Δ*Fgmvp1* | *FgMVP1* deletion mutant of PH-1 | | This study |
| Δ*Fgmvp1-C* | Δ*Fgmvp1* transformant expressing FgMvp1-GFP  construct | | This study |
| Δ*Fgvps5-C* | Δ*Fgvps5* transformant expressing FgVps5-GFP  construct | | This study |
| Δ*Fgvps17-C* | Δ*Fgvps17* transformant expressing FgVps17-GFP  construct | | This study |
| PH-1*+*FgSnc1-GFP | PH-1 strain expressing FgSnc1-GFP construct | | This study |
| PH-1*+*FgExo84-GFP | PH-1 strainexpressing FgExo84-GFP construct | | This study |
| PH-1*+*FgDnfA-GFP | PH-1 strain expressing FgDnfA-GFP construct | | This study |
| PH-1*+*FgDnfB-GFP | PH-1 strain expressing FgDnfB-GFP construct | | This study |
| Δ*Fgbem1+*FgSnc1-GFP | Δ*Fgbem1* transformant expressing  FgSnc1-GFP construct | | This study |
| Δ*Fgbem1+*FgExo84-GFP | Δ*Fgbem1* transformant expressing  FgExo84-GFP construct | | This study |
| Δ*Fgbem1+*FgDnfA-GFP | Δ*Fgbem1* transformant expressing  FgDnfA-GFP construct | | This study |
| Δ*Fgbem1+*FgDnfB-GFP | Δ*Fgbem1* transformant expressing  FgDnfB-GFP construct | | This study |
| Δ*Fgsnx4-C* | Δ*Fgsnx4* transformant expressing  FgSnx4-GFP construct | | Zheng et al. (2018) |
| Δ*Fgsnx41-C* | Δ*Fgsnx41* transformant expressing  FgSnx41-GFP construct | | Zheng et al. (2018) |
| Δ*Fgrho4* | *FgRHO4* deletion mutant of PH-1 | | Zhang et al. (2013) |
| **B. Plasmids used in this study.** | | | |
| **Clone** | **Description** | | |
| pFgBem1-GFP | For expression of FgBem1:GFP, cloned in *Kpn*I-*Hind*III sites of  pKNT-GFP. Ampicillin and Neomycin resistance. | | |
| pFgBem1ΔSH3-1-GFP | For expression of FgBem1ΔSH3-1:GFP, cloned in *Kpn*I-*Hind*III sites of  pKNT-GFP. Ampicillin and Neomycin resistance. | | |
| pFgBem1ΔSH3-2-GFP | For expression of FgBem1ΔSH3-2:GFP, cloned in *Kpn*I-*Hind*III sites of  pKNT-GFP. Ampicillin and Neomycin resistance. | | |
| pFgBem1ΔPX-GFP | For expression of FgBem1ΔPX:GFP, cloned in *Kpn*I-*Hind*III sites of  pKNT-GFP. Ampicillin and Neomycin resistance. | | |
| pFgBem1ΔPB1-GFP | For expression of FgBem1ΔPB1:GFP, cloned in *Kpn*I-*Hind*III sites of  pKNT-GFP. Ampicillin and Neomycin resistance. | | |
| pFgSnx3-GFP | For expression of FgSnx3:GFP, cloned in *Kpn*I-*Hind*III sites of  pKNT-GFP. Ampicillin and Neomycin resistance. | | |
| pFgSpo14-GFP | For expression of FgSpo14:GFP, cloned in KpnI-HindIII sites of  pKNT-GFP. Ampicillin and Neomycin resistance. | | |
| pFgYpt35-GFP | For expression of FgYpt35:GFP, cloned in *Kpn*I-*Hind*III sites of  pKNT-GFP. Ampicillin and Neomycin resistance. | | |
| pFgSnx19-RFP | For expression of FgSnx19:GFP, cloned in *Kpn*I-*Hind*III sites of  pKNT-GFP. Ampicillin and Neomycin resistance. | | |
| pFgMdm1-GFP | For expression of FgMdm1:GFP, cloned in *Kpn*I-*Hind*III sites of  pKNT-GFP. Ampicillin and Neomycin resistance. | | |
| pFgMvp1-GFP | For expression of FgMvp1:GFP, cloned in *Kpn*I-*Hind*III sites of  pKNT-GFP. Ampicillin and Neomycin resistance. | | |
| pFgVps5-GFP | For expression of FgVps5:GFP, cloned in *Kpn*I-*Hind*III sites of  pKNT-GFP. Ampicillin and Neomycin resistance. | | |
| pFgVps17-GFP | For expression of FgVps17:GFP, cloned in *Kpn*I-*Hind*III sites of  pKNT-GFP. Ampicillin and Neomycin resistance. | | |
| pFgExo84-GFP | For expression of FgExo84:GFP, cloned in *Kpn*I-*Hind*III sites of  pKNT-GFP. Ampicillin and Neomycin resistance. | | |
| pFgYkr078-GFP | For expression of FgYkr078:GFP, cloned in *Kpn*I-*Hind*III sites of  pKNT-GFP. Ampicillin and Neomycin resistance. | | |
| pFgYpr097-GFP | For expression of FgYpr097:GFP, cloned in *Kpn*I-*Hind*III sites of  pKNT-GFP. Ampicillin and Neomycin resistance. | | |
| pFgSnx4-GFP | For expression of FgSnx4:GFP, obtained from Zheng et al., 2018. | | |
| pFgSnx41-GFP | For expression of FgSnx41:GFP, obtained from Zheng et al., 2018. | | |
| pLifeAct-RFP | For expression of Lifeactin:RFP, obtained from Zhang et al., 2016. | | |
| pFgDnfA-GFP | For expression of FgDnfA:GFP, obtained from Zhang et al., 2019. | | |
| pFgDnfB-GFP | For expression of FgDnfB:GFP, obtained from Zhang et al., 2019. | | |
| pFgSnc1-GFP | For expression of FgSnc1:GFP, obtained from Zheng et al., 2018. | | |
| C. **PCR primers used in this study** | | | |
| **Primers** | **Sequence(5’-3’)** | **Application** | |
| FgBEM1-AF | AGATTGAAGTTGGCTAAGGGC | *FgBEM1* deletion and probe | |
| FgBEM1-AR | TTGACCTCCACTAGCTCCAGCCAAGCCACCGAAGCGTTGTGGAAGT |  |  |
| FgBEM1-BF | GAATAGAGTAGATGCCGACCGCGGGTTTTGCATCCCTTCAATACCCG |  |  |
| FgBEM1-BR | GCTTTCCACCACCACCTTCA |  |  |
| FgBEM1-OF | GTGATAAGGACAAAGGACCAGT | *∆Fgbem1* mutant screen | |
| FgBEM1-OR | AGCAGGGCAATCTGGAAAT |  |  |
| FgBEM1-UA | CAAGCGGGAGGTCTGTATGC |  |  |
| H853 | GACAGACGTCGCGGTGAGTT |  |  |
| FgSPO14-AF | CACAACTGGACGAGGCTAAG | *FgSPO14* deletion and probe | |
| FgSPO14-AR | TTGACCTCCACTAGCTCCAGCCAAGCCAAGGTGGTGACGAACTAAAACA |  |  |
| FgSPO14-BF | GAATAGAGTAGATGCCGACCGCGGGTTCCATACGACTGGCTCCTCAC |  |  |
| FgSPO14-BR | GCTGTATCAAACGAGTCTTCCA |  |  |
| FgSPO14-OF | ATGGCAACGAGAAGACAACG | *∆Fgspo14* mutant screen | |
| FgSPO14-OR | AAGCAGAAAAGGCAAGGGTC |  |  |
| FgSPO14-UA | ATCTTGGACAGACTGCCCTCA |  |  |
| FgSNX3-AF | TCATTGGGCTTACGCAGGTC | *FgSNX3* deletion and probe | |
| FgSNX3-AR | TTGACCTCCACTAGCTCCAGCCAAGCCGAGGCTCGTGGTGATGTTTTG |  |  |
| FgSNX3-BF | GAATAGAGTAGATGCCGACCGCGGGTTAAAAGCAAGTTCTGGTTCGG |  |  |
| FgSNX3-BR | GGTTCATCTATCGGCTGGTG |  |  |
| FgSNX3-OF | GGCCTCCCGAGAACTTCCTA | *∆Fgsnx3* mutant screen | |
| FgSNX3-OR | GGTCTGCGAGCGTGTCAAT |  |  |
| FgSNX3-UA | CAGCCCGTCGCTTACCCTA |  |  |
| FgYPT35-AF | ATTGGTCGCTGGAAGTGTAAG | *FgYPT35* deletion and probe | |
| FgYPT35-AR | TTGACCTCCACTAGCTCCAGCCAAGC  CATGCGTCGGTAGAAATGGTC |  |  |
| FgYPT35-BF | GAATAGAGTAGATGCCGACCGCGGGTTGTAGTCCAGATTCACCCAGTC |  |  |
| FgYPT35-BR | CGTAGTCAAGCCAGTAAAAGTA |  |  |
| FgYPT35-OF | CAACGGTAGTGCGACAAATA | *∆Fgypt35* mutant screen | |
| FgYPT35-OR | CACGTCGTAAAATGCAGATC |  |  |
| FgYPT35-UA | GGACTCCGAATATCGCAGGTG |  |  |
| FgYPR097-AF | ATTTCGATGCCATCATTACTC | *FgYPR097* deletion and probe | |
| FgYPR097-AR | TTGACCTCCACTAGCTCCAGCCAAGCCGCATTTATCGCTCTGGACAC |  |  |
| FgYPR097-BF | GAATAGAGTAGATGCCGACCGCGGGTTCTTATGATGAAAGAGCAGGATTGA |  |  |
| FgYPR097-BR | CTTGGCTCGTAGAGGACCGT |  |  |
| FgYPR097-OF | GAGAGTTGGAGCATTTTGTTG | *∆Fgypr097* mutant screen | |
| FgYPR097-OR | GCGTTGTAGAGTCGTTGGAT |  |  |
| FgYPR097-UA | GCATACGCCGAAGCATAGAGG |  |  |
| FgYKR078-AF | GATTGCCAACAAGAACCACCAG | *FgYKR078* deletion and probe | |
| FgYKR078-AR | TTGACCTCCACTAGCTCCAGCCAAGCCATGCCAGTTATTATTTAGGTAGGTATCG |  |  |
| FgYKR078-BF | GAATAGAGTAGATGCCGACCGCGGGTTTTCAAGGACTGGATAGAGGCAATA |  |  |
| FgYKR078-BR | TCTCCTTCCAATTCAGCCACC |  |  |
| FgYKR078-OF | CCGTGGGAACGAGAAAGGTGT | *∆Fgykr078* mutant screen | |
| FgYKR078-OR | GACTCGTTTGGCTTACTGGTCTTTAT |  |  |
| FgYKR078-UA | AGGACCTCTGGGAAGCTATTGG |  |  |
| FgSNX19-AF | GAAGGGGTTTCCATCGTCAT | *FgSNX19* deletion and probe | |
| FgSNX19-AR | TTGACCTCCACTAGCTCCAGCCAAGCCAATGGTGAATGTTCTCGGTAGG |  |  |
| FgSNX19-BF | GAATAGAGTAGATGCCGACCGCGGGTTAGAAAACGACCTACGGCAACG |  |  |
| FgSNX19-BR | TCGGTCAACCAAGCCACAAT |  |  |
| FgSNX19-OF | AGCCTAACTGGGATTACTGG | *∆Fgsnx19* mutant screen | |
| FgSNX19-OR | CTGAAAGTGCGGTGTATTGT |  |  |
| FgSNX19-UA | AGCGAGATGCCCTATTTGGA |  |  |
| FgMDM1-AF | GGTTGCGGTTTCGGAGGTCT | *FgMDM1* deletion and probe | |
| FgMDM1-AR | TTGACCTCCACTAGCTCCAGCCAAGCCAGGCTATGAAGGCGGCGAGT |  |  |
| FgMDM1-BF | GAATAGAGTAGATGCCGACCGCGGGTTTTTCAACTCCTCCGTAGTATCC |  |  |
| FgMDM1-BR | ATCCGTCAAGACAAGAACCA |  |  |
| FgMDM1-OF | ATCAACACCGCAACCAGAAG | *∆Fgmdm1* mutant screen | |
| FgMDM1-OR | TCGCCGAGGAAAGTCAAGA |  |  |
| FgMDM1-UA | ATCACAAGAAGAATGACGGGAAC |  |  |
| FgMVP1-AF | GTCCTCACCCGATACTACACCA | *FgMVP1* deletion and probe | |
| FgMVP1-AR | TTGACCTCCACTAGCTCCAGCCAAGCCAAAGAGCCCAGAACCACCAC |  |  |
| FgMVP1-BF | GAATAGAGTAGATGCCGACCGCGGGTTTACCCCTCATTCTTTAGTTTCG |  |  |
| FgMVP1-BR | CTGCCTGGTTCATCATCGTA |  |  |
| FgMVP1-OF | AGACCCCGAAGACGACCCAT | *∆Fgmvp1* mutant screen | |
| FgMVP1-OR | TCTCGCCAGCCTTGACCATA |  |  |
| FgMVP1-UA | GAGTTGGTCGTGGTCCTTCG |  |  |
| FgBEM1-CF | agggaacaaaagctgggtaccAGTTAGTCCTTGGTATGGTCCGTC | For generation of pFgBem1-GFP,  pFgSnx3-GFP,  pFgSpo14-GFP,  pFgMdm1-GFP,  pFgYpt35-GFP, pFgSnx19-GFP, pFgMvp1-GFP,  pFgVps5-GFP,  pFgVps17-GFP,  pFgExo84-GFP,  pFgYpr097-GFP and  pFgYkr078-GFP | |
| FgBEM1-CR | gcccttgctcaccataagcttTACCGCCTCCACGTAGAGAAGC |  |  |
| FgSNX3-CF | agggaacaaaagctgggtaccAGCAAGCACGCAAGCAGC |  |  |
| FgSNX3-CR | gcccttgctcaccataagcttCCAAGCGTTGCGATCCCA |  |  |
| FgSPO14-CF | agggaacaaaagctgggtaccTGCCCAACTCTGTAGGTTTCGT |  |  |
| FgSPO14-CR | gcccttgctcaccataagcttGTTGCTGTAACTTGTTAGCATGACTG |  |  |
| FgMDM1-CF | agggaacaaaagctgggtaccGCCTACATTGCCACGAGCC |  |  |
| FgMDM1-CR | gcccttgctcaccataagcttTCGAATGTCGTACTTCAACCTTGAG |  |  |
| FgYPT35-CF | agggaacaaaagctgggtaccTTCTGCTACCCACTAGAATGTCT |  |  |
| FgYPT35-CR | gcccttgctcaccataagcttTGCAAAGAGAAACTCTTTGAGTACA |  |  |
| FgSNX19-CF | agggaacaaaagctgggtaccACTGAGCGAGATGCCCTATTTG |  |  |
| FgSNX19-CR | gcccttgctcaccataagcttGTGTGTTACAATTCGAACAACTTGG |  |  |
| FgMVP1-CF | agggaacaaaagctgggtaccGACGCACCGTTCCGATACC |  |  |
| FgMVP1-CR | gcccttgctcaccataagcttGTCACCCAGCGGCATGCC |  |  |
| FgVPS5-CF | agggaacaaaagctgggtaccAAACGGTCGGTACTCATACTAACA |  |  |
| FgVPS5-CR | gcccttgctcaccataagcttATCCGAGTCTTCGTCTATCCTTG |  |  |
| FgVPS17-CF | agggaacaaaagctgggtaccCGGCTTCCTCCTTGGCTTT |  |  |
| FgVPS17-CR | gcccttgctcaccataagcttGAAAGTGCTGGCGGCCA |  |  |
| FgEXO84-CF | agggaacaaaagctgggtaccAGGATAGACCTTGAGACCCTTGT |  |  |
| FgEXO84-CR | gcccttgctcaccataagcttCGATAAGCCCAACCCCACC |  |  |
| FgYPR097-CF | agggaacaaaagctgggtaccCGCATACTCGTTCTGGTCAGG |  |  |
| FgYPR097-CR | gcccttgctcaccataagcttATTAGACAGAACTTGTCGCAGC |  |  |
| FgYKR078-CF | agggaacaaaagctgggtaccGCTGAGGCCAAGAACAACCC |  |  |
| FgYKR078-CR | gcccttgctcaccataagcttAGTCTCTTGCAATATCTTCCGAA |  |  |
| FgBEM1-CF | agggaacaaaagctgggtaccAGTTAGTCCTTGGTAT  GGTCCGTC | For generation of  pFgBem1ΔSH3-1-GFP、pFgBem1ΔSH3-2-GFP、  pFgBem1ΔPX-GFP and pFgBem1ΔPB1-GFP  constructs | |
| FgBEM1-CRNSH3-1 | GTGCACTATCTCGCTCGGTTCGCTTGG  GAGGAACAATGGCGA |  |  |
| FgBEM1-CNSH3-1F | TCGCCATTGTTCCTCCCAAGCGAACCGAGCGAGATAGTGCAC |  |  |
| FgBEM1-CNSH3-1R | AATCGCCTTATCGCTTGCCATCATGGCACCCGTCTTTCCG |  |  |
| FgBEM1-CNSH3-2F | CGGAAAGACGGGTGCCATGATGGCAAGCGATAAGGCGATT |  |  |
| FgBEM1-CNPX-R | GGGTCGATCTCGTAGTCTCCTTCGAGAGGAGCATGGAGAGGAGCA |  |  |
| FgBEM1-CPX-F | TGCTCCTCTCCATGCTCCTCTCGAAGGAGACTACGAGATCGACCC |  |  |
| FgBEM1-CR2 | gcccttgctcaccataagcttTACCGCCTCCACGTAGAGAAGC |  |  |
| FgBEM1-CNPB1-R | gcccttgctcaccataagcttTACCGCCTCCATTGCGGATGGTGGTTGTG |  |  |

**Reference:**

1. Cuomo CA, Güldener U, Xu JR, Trail F, Turgeon BG, Di Pietro A, Walton JD, Ma LJ, Baker SE, Rep M, Adam G, Antoniw J, Baldwin T, Calvo S, Chang YL, Decaprio D, Gale LR, Gnerre S, Goswami RS, Hammond-Kosack K, Harris LJ, Hilburn K, Kennell JC, Kroken S, Magnuson JK, Mannhaupt G, Mauceli E, Mewes HW, Mitterbauer R, Muehlbauer G, Münsterkötter M, Nelson D, O'Donnell K, Ouellet T, Qi W, Quesneville H, Roncero MI, Seong KY, Tetko IV, Urban M, Waalwijk C, Ward TJ, Yao J, Birren BW, Kistler HC. 2007. The Fusarium graminearum genome reveals a link between localized polymorphism and pathogen specialization. Science 317:1400-2.
2. Li B, Dong X, Zhao R, Kou R, Zheng X, Zhang H. 2019. The t-SNARE protein FgPep12, associated with FgVam7, is essential for ascospore discharge and plant infection by trafficking Ca2+ ATPase FgNeo1 between Golgi and endosome/vacuole in *Fusarium graminearum*. PLoS Pathog 15:e1007754.
3. Li B, Liu L, Li Y, Dong X, Zhang H, Chen H, Zheng X, Zhang Z. 2017. The FgVps39-FgVam7-FgSso1 Complex Mediates Vesicle Trafficking and Is Important for the Development and Virulence of *Fusarium graminearum*. Mol Plant Microbe Interact 30:410-422.
4. Liu Z, Wang Z, Huang M, Yan L, Ma Z, Yin Y. 2017. The FgSsb-FgZuo-FgSsz complex regulates multiple stress responses and mycotoxin production via folding the soluble SNARE Vam7 and β2-tubulin in *Fusarium graminearum*. Environ Microbiol 19:5040-5059.
5. Zhang C, Wang Y, Wang J, Zhai Z, Zhang L, Zheng W, Zheng W, Yu W, Zhou J, Lu G, Shim WB, Wang Z. 2013. Functional characterization of Rho family small GTPases in *Fusarium graminearum*. Fungal Genet Biol 61:90-9.
6. Zhang H, Li B, Fang Q, Li Y, Zheng X, Zhang Z. 2016. SNARE protein FgVam7 controls growth, asexual and sexual development, and plant infection in *Fusarium graminearum*. Mol Plant Pathol 17:108-19.
7. Zhang J, Yun Y, Lou Y, Abubakar YS, Guo P, Wang S, Li C, Feng Y, Adnan M, Zhou J, Lu GD, Zheng W. 2019. FgAP-2 complex is essential for pathogenicity and polarised growth and

regulates the apical localisation of membrane lipid flippases in Fusarium graminearum. Cell Microbiol 21:e13041.

1. Zheng W, Zheng H, Zhao X, Zhang Y, Xie Q, Lin X, Chen A, Yu W, Lu G, Shim WB, Zhou J, Wang

Z. 2016. Retrograde trafficking from the endosome to the trans-Golgi network mediated by the retromer is required for fungal development and pathogenicity in *Fusarium graminearum*. New Phytol 210:1327-43.

1. Zheng W, Lin Y, Fang W, Zhao X, Lou Y, Wang G, Zheng H, Liang Q, Abubakar YS, Olsson S, Zhou J, Wang Z. 2018. The endosomal recycling of FgSnc1 by FgSnx41-FgSnx4 heterodimer is essential for polarized growth and pathogenicity in *Fusarium graminearum*. New Phytol 219:654-671.
